# A Fur family protein BosR is a novel RNA-binding protein that controls *rpoS* RNA stability in the Lyme disease pathogen

**DOI:** 10.1093/nar/gkae114

**Published:** 2024-02-15

**Authors:** Sajith Raghunandanan, Raj Priya, Fuad Alanazi, Meghan C Lybecker, Paula Jean Schlax, X Frank Yang

**Affiliations:** Department of Microbiology and Immunology, Indiana University School of Medicine, Indianapolis, IN 46202, USA; Department of Microbiology and Immunology, Indiana University School of Medicine, Indianapolis, IN 46202, USA; Department of Microbiology and Immunology, Indiana University School of Medicine, Indianapolis, IN 46202, USA; Department of Clinical Laboratory Sciences, College of Applied Medical Sciences, King Saud University, Riyadh 12372, Saudi Arabia; Bacterial Diseases Branch, Division of Vector-Borne Diseases, National Center for Emerging and Zoonotic Infectious Diseases, Center for Disease Control and Prevention, Fort Collins, CO, USA; Department of Chemistry and Biochemistry, Bates College, Lewiston, ME, USA; Department of Microbiology and Immunology, Indiana University School of Medicine, Indianapolis, IN 46202, USA

## Abstract

The σ^54^-σ^S^ sigma factor cascade plays a central role in regulating differential gene expression during the enzootic cycle of *Borreliella burgdorferi*, the Lyme disease pathogen. In this pathway, the primary transcription of *rpoS* (which encodes σ^S^) is under the control of σ^54^ which is activated by a bacterial enhancer-binding protein (EBP), Rrp2. The σ^54^-dependent activation in *B. burgdorferi* has long been thought to be unique, requiring an additional factor, BosR, a homologue of classical Fur/PerR repressor/activator. However, how BosR is involved in this σ^54^-dependent activation remains unclear and perplexing. In this study, we demonstrate that BosR does not function as a regulator for *rpoS* transcriptional activation. Instead, it functions as a novel RNA-binding protein that governs the turnover rate of *rpoS* mRNA. We further show that BosR directly binds to the 5′ untranslated region (UTR) of *rpoS* mRNA, and the binding region overlaps with a region required for *rpoS* mRNA degradation. Mutations within this 5′UTR region result in BosR-independent RpoS production. Collectively, these results uncover a novel role of Fur/PerR family regulators as RNA-binding proteins and redefine the paradigm of the σ^54^–σ^S^ pathway in *B. burgdorferi*.

## Graphical Abstract

**Graphical Abstract:**
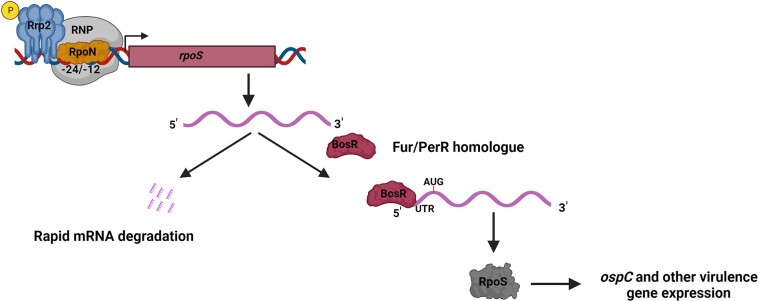

## Introduction

Lyme disease is the most prevalent arthropod-borne infection in the United States, Europe, and Asia (1). The causative agent, *Borreliella* (or *Borrelia*) *burgdorferi* (*Bb*), is maintained in nature through an enzootic cycle involving a tick vector and a mammalian host (2). To adapt to its environment and persist in each phase of its enzootic cycle, *B. burgdorferi* undergoes dramatic regulation of its gene expression (3–7). Over the past two decades, several regulators and signaling pathways governing differential gene expression in *B. burgdorferi* have been identified that (3–7). Notably, the σ^N^–σ^S^ (RpoN-RpoS) alternative σ factor cascade has been the most studied gene regulatory pathway (8). In this pathway, the alternative sigma factor RpoN controls the production of the second alternative sigma factor RpoS, which, in turn, functions as a global regulator. RpoS activates the transcription of many virulence genes essential for transmission and vertebrate host infection while repressing expression of genes required for spirochete survival in the tick vector (6,9–11).

In *B. burgdorferi*, *rpoS* is primarily transcribed from a RpoN (σ^54^)-type promoter, critical for the enzootic cycle (8,10–12). *rpoS* can also be transcribed from a σ^70^-type promoter, producing a long transcript whose role in *B. burgdorferi* pathogenesis remains to be determined (13). In the *B. burgdorferi* genome, *rpoS* is the sole gene identified as having a σ^54^-type promoter thus far (14). RpoN directly binds to the *rpoS* promoter and activates *rpoS* transcription (15–17). In bacterial kingdoms, σ^54^-dependent transcriptional activation is well-established, requiring a unique transcriptional activator, the bacterial Enhancer-Binding Protein (bEBP) (18–20). Rrp2 is the only bEBP present in the *B. burgdorferi* genome (21). Rrp2 is a homologue of nitrogen regulator NtrC, a well characterized bEBP in *E. coli and Salmonella*. It has been demonstrated to be essential for *rpoS* transcription (6,22–24). Hence, the σ^N^–σ^S^ alternative σ factor cascade also has been called as Rrp2–RpoN–RpoS pathway (6,21,25,26).

It has been long believed that the mechanism underlying the σ^54^-dependent activation of *rpoS* transcription in *B. burgdorferi* is unique: it requires not only Rrp2 but also another transcriptional activator BosR (Borrelia Oxidative Stress Regulatory Protein), a homolog of the Fur/PerR protein family (16,27–32). Major evidence supporting BosR as the transcriptional activator of *rpoS* includes that (i) inactivation of *bosR* abolished *rpoS* expression (29,31); (ii) BosR binds to the σ^54^-dependent promoter region of *rpoS in vitro* (16,33); (iii) IPTG-inducible *rpoS* expression produced a dose-dependent *rpoS* mRNA production in a *bosR* deletion mutant (16,34). However, the mechanism of how BosR may activate σ^54^-dependent gene transcription has remained a mystery and is perplexing, particularly as the mechanism of σ^54^-dependent activation is well-established in other bacteria, where bEBP is sufficient for σ^54^-dependent transcriptional activation both *in cellulo* and *in vitro* (35–37). In fact, direct evidence demonstrating that BosR functions as a transcriptional activator for *rpoS* remains lacking, and the functional significance of the BosR binding sites identified *in vitro* for *rpoS* expression also has not been investigated *in cellulo*.

In this study, we first employed a transcription reporter system to examine the role of BosR in the transcriptional activation of *rpoS* in *B. burgdorferi*. Our findings challenge the current dogma, demonstrating that BosR is not required for σ^54^-dependent transcription activation of *rpoS*. Instead, we reveal that BosR regulates *rpoS* post-transcriptionally by controlling the turnover rate of *rpoS* mRNA. Furthermore, we establish that BosR is a novel RNA-binding protein that directly binds to the 5′ UTR region of *rpoS* mRNA, preventing mRNA degradation. Thus, this finding redefines the paradigm of the σ^N^-σ^S^ sigma factor cascade in *B. burgdorferi*. Additionally, this study uncovers a novel role for the Fur protein family, i.e. functions as RNA-binding proteins.

## Materials and methods

### *B. burgdorferi* strains and culture conditions

Low-passage, *B*. *burgdorferi* strains AH130 and 5A14 strains (a gift from Drs H. Kawabata and S. Norris, University of Texas Health Science Center at Houston) were used in this study. Spirochetes were cultivated in Barbour-Stoenner-Kelly (BSK-II) medium supplemented with 6% normal rabbit serum (Pel-Freez Biologicals, Rogers, AR) at 37°C with 5% CO_2_ (38). At the time of growth, appropriate antibiotics were added to the cultures with the following final concentrations: 300 μg/ml for kanamycin, 50 μg/ml for streptomycin, and 50 μg/ml for gentamicin. All the constructed plasmids were maintained in *E*scherichia *coli* strain DH5α. The antibiotic concentrations used for *E*. *coli* selection were as follows: streptomycin, 50 μg/ml; gentamicin, 15 μg/ml and rifampicin, 50 μg/ml, respectively. All *B*. *burgdorferi* strains and plasmids used in this study are listed in the Supplementary Table S1 and S2.

### Construction of the luciferase reporter driven by various lengths of the *rpoS* promoter

For the construction of the shuttle plasmid BS1^+^/BS2^+^-*luc*, a 250 bp upstream region of *rpoS* ORF containing both BS1, BS2 –24/–12 *rpoS* promoter, and the 5′UTR region) was PCR amplified from *B*. *burgdorferi* DNA using specific set of primers (Supplementary Table S3). The amplified fragment was then cloned into the upstream of a promoter less *luc* on a pBSV2-deried shuttle vector pJD48 utilizing *Nde*I and *Nco*I restriction enzymes. Similarly, for the construction of BS1^−^/BS2^+^-*luc* and BS1^−^/BS2^−^-*luc* reporter plasmids, a 95 and a 75 bp fragment were PCR amplified and ligated to the upstream of promoterless *luc* at the *Nde*I and *Nco*I restriction sites, respectively. The resulting plasmids were confirmed by sequencing and were transformed into the wild-type *B. burgdorferi* strain B31M and the *bosR* mutant (OY10H) (31).

### Construction of *B*. *burgdorferi* strains carrying the *rpoS* promoter-driven *gfp* in the chromosome

To replace the chromosomal copy of *rpoS* with a codon optimized *B*. *burgdorferi gfp*, we generated a suicidal plasmid pSR074. Firstly, a 1.5 kb downstream region of *rpoS* (810988–812488) was PCR amplified form the *B*. *burgdorferi* genome using a specific set of primers (Supplementary Table S3). The fragment was then inserted into the *Cla*I restriction site downstream of an *aadA* streptomycin-resistant marker within the suicide vector pMP026, resulting in the suicidal plasmid pSR028. For constructing the *rpoSp-gfp* fusion, a 1.5 kb up stream region of the *rpoS* start codon (813240–814740 bp containing both BS1, BS2, –24/–12 *rpoS* promoter and 5′UTR respectively) and a codon optimized *B*. *burgdorferi gfp* ORF were PCR amplified from the *B*. *burgdorferi* genome and shuttle plasmid pTM61 using specific sets of over lapping primers (Supplementary Table S3). The PCR fragments were assembled on to BamHI digested pSR028 using the NEBuilder^®^ Assembly Tool mix. The resulting streptomycin resistant plasmid, pSR074, was sequenced and was transformed into the wild-type strain, the *rpoN* mutant, and the *bosR* mutants (39,40). The transformants were selected based on streptomycin resistance and replacements were further confirmed by PCR and sequencing.

### Construction of shuttle plasmids carrying the *rpoS* gene with various mutations within the 5′ UTR region

For all mutagenesis studies, the *rpoS* coding sequence (CDS) along with its minimal –24–12 promoter containing either the native or truncated 5′UTR sequences was PCR amplified from the *B*. *burgdorferi* genome using specific sets of primers (Supplementary Table S3). Subsequently, the PCR fragments were ligated onto pBSV2B vector using *Sac*I and *Sph*I restriction sites. The resulting plasmids were selected based on rifampicin resistance in *E. coli* (50 μg/ml). After sequencing, individual plasmids were then transformed into the *rpoS* or *bosR* mutant, and transformants were selected based on blasticidin resistance (40 μg/ml).

### RNA decay assays

Measurements of *rpoS* mRNA decay were performed as described previously (41). Briefly, both the wild-type strain B31M and the *bosR* mutant were grown to stationary phase at 37°C in BSK II pH (7.0) medium in 200 ml cultures. Before the addition of actinomycin D (Millipore Sigma), 20 ml of culture was taken and centrifuged at 8000 g for 10 min. The pellets were frozen for further use. For the remaining 180 ml culture, actinomycin D was added to a final concentration of 150 μg/ml. Aliquots from each culture were collected at time points of 0.5, 1, 5, 15, 30, 45, 60, 120 and 240 min after actinomycin treatment. All pellets were washed twice with cold PBS. RNA was extracted from all pellets using RNeasy mini kit (Qiagen, Valencia, CA) according to the manufacturer's protocols (42), followed by on-column digestion using RNase-free DNase I (Promega, Madison, WI). cDNA was synthesized from isolated RNAs using the SuperScript III reverse transcriptase with random primers (Invitrogen, Carlsbad, CA), followed by qRT-PCR analysis using PowerUp SYBR Green Master Mix (Thermo Fisher Scientific) on a QuantStudio™ 3 Real-Time PCR thermocycler. Decay data were analyzed and the remaining fraction of the *rpoS* RNA (*f*) was calculated using the formula *f* = 2^(^*^CT^*^ref −^*^CT^*^)^, where *C_T_*_ref_ is the *C_T_* value determined for the mRNA from an antibiotic-free culture, and *C_T_* is the value for the mRNA purified from culture at a given time after antibiotic addition. The remaining fraction of *rpoS* RNA is plotted on a logarithmic scale.

### RNA immunoprecipitation

RNA immunoprecipitation was performed using the BbYY028 *B*. *burgdorferi* strain (wild-type *B. burgdorferi* expressing (*lacp-bosR-HA*) shuttle plasmid). After culturing the recombinant *B*. *burgdorferi* in BSK II pH(7.5) medium at 37°C in the presence of IPTG (100 μg/ml), RNA immunoprecipitation was performed according to previously published protocol with minor modifications (43). In brief, *B*. *burgdorferi* cultures were fixed in 1% formaldehyde for 15 min at room temperature on a shaker. After centrifugation, pellets were washed twice with ice cold PBS, and then resuspended in lysis buffer containing 20 mM Tris–HCl (pH 7.5), 150 mM NaCl, 2.5 mM MgCl2, 1% Triton X-100, 10% glycerol, 0.5 mM DTT, 0.1% sodium deoxycholate, 1x protease inhibitors cock tail and RNase inhibitor (RNASIN, Promega, 50 U/500 μl), followed by extensive sonication to fragment nucleic acids. DNase-treated lysates were incubated with anti-HA, anti-YebC or anti-mouse IgG at 4°C overnight. The reaction mixtures were centrifuged at maximum speed in a microcentrifuge for 15 min and the supernatants were collected. Fifty microliters of protein A/G slurry (equilibrated in lysis buffer containing 1 mg/ml bovine serum albumin and RNase inhibitor) were added to the supernatant and incubated for 5 hrs at 4°C. The immunoprecipitated complexes were then washed three times in wash buffer containing 20 mM Tris–HCl (pH 7.5), 150 mM NaCl, 2.5 mM MgCl2, 0.2% Triton X-100, 10% glycerol, 0.5 mM DTT, 1x protease inhibitors cock tail and RNase inhibitor (RNASIN, Promega; 50 U/500 μl), and once in dilution buffer containing 20 mM Tris–HCl (pH 7.5), 150 mM NaCl, 2.5 mM MgCl2, 10% glycerol, 1× protease inhibitors cock tail and RNase inhibitor (RNASIN, Promega; 50 U/500 μl). The immunoprecipitated complexes were resuspended in 50 μl of elution buffer containing 1% SDS and 100 mM sodium bicarbonate supplemented with RNase inhibitor. Proteinase K was added and incubated at 55°C for 30 mins to elute protein and RNAs from the beads. The beads were resuspended in TRIzol reagent (Sigma Aldrich). The RNA was then extracted using RNeasy mini kit (Qiagen, Valencia, CA) according to the manufacturer's protocols (42), followed by on-column digestion using RNase-free DNase I (Promega, Madison, WI). Purified RNAs were subjected to cDNA synthesis using the SuperScript III reverse transcriptase with random primers (Invitrogen, Carlsbad, CA), followed by qRT-PCR analysis using PowerUp SYBR Green Master Mix (Thermo Fisher Scientific) on a QuantStudio™ 3 Real-Time PCR thermocycler. The relative levels of the corresponding RNA species in immunoprecipitated samples using anti-HA or anti-YebC were compared to those using IgG (normalized to 1.0).

### Immunoblotting

Spirochetes from mid-log or stationary phase-grown cultures were harvested by centrifuging at 8000 × g for 10 min, followed by three washes with PBS (pH 7.4) at 4°C. Pellets were suspended in SDS buffer containing 50 mM Tris–HCl (pH 8.0), 0.3% sodium dodecyl sulfate (SDS) and 10 mM dithiothreitol (DTT). Cell lysates (10^8^ cells per lane) were separated by 12% SDS-polyacrylamide gel electrophoresis (PAGE) and transferred to nitrocellulose membranes (GE-Healthcare, Milwaukee, WI). Membranes were blotted with mouse monoclonal antibody of anti-BosR (1:4000 dilution), anti-FlaB (1:1000 dilution), anti-OspC (1:1000 dilution), anti-HA (1:1000), or anti-RpoS (1:100 dilution) (29,42,44), followed by anti-mouse IgG-HRP secondary antibody (1:1000; Santa Cruz Biotechnology). Detection of horseradish peroxidase activity was performed using the enhanced chemiluminescence method (Thermo Pierce ECL Western Blotting Substrate) with subsequent exposure to X-ray film.

### Quantitative real time (q-PCR) PCR analyses

RNA samples were extracted from *B. burgdorferi* cultures using the RNeasy mini kit (Qiagen, Valencia, CA) according to the manufacturer's protocols (42), followed by on-column treatment with RNase-free DNase I treatment Promega, Madison, WI). The quality of DNA-free RNA was confirmed by PCR amplification of *flaB* of *B*. *burgdorferi*. The cDNA was synthesized using the SuperScript III reverse transcriptase with random primers (Invitrogen, Carlsbad, CA). All the primers used for qPCR (Supplementary Table S3) were designed using Primer BLAST software. The cycling conditions were set as follows – an initial denaturation of 94°C for 5 min; 35 cycles of denaturation at 94°C for 30 s, primer annealing at 59°C for 30 s, and extension at 72°C for 40 s, followed by a melt curve analysis. All reactions were carried out in 3 independent experiments using an QuantStudio™ 3 Real-Time PCR thermocycler and were analyzed using QuantStudio™ 3 Real-Time PCR software. Calculations of relative levels of transcript were normalized with the *flaB* transcript levels as per previous reports (42).

### *In vitro* transcription for generating ^32^P-labelled RNA probes

All *in vitro* transcription reactions were performed using the MEGAscript® Kit (life technologies) by following the manufacturer's instruction. The transcripts were prepared using T7 RNA polymerase and a DNA template driven by a T7 promoter constructed by PCR amplification using a respective set of oligos containing a consensus sequence TAATACGACTCACTATAGGG (Supplementary Table S4). ^32^P labelled RNA was transcribed in a reaction mixture containing 0.75 mM UTP, 0.75 mM ATP, 0.75 mM CTP and 0.75 mM GTP along with 1X reaction and enzyme mix. Twenty-five μCi of α-^32^P UTP was added to the final reaction mixture and the *in vitro* transcription was achieved by incubating the mixture at 37°C for 4 h. RNA was purified using the Monarch® RNA Cleanup Kit (New England Biolabs). The transcribed RNAs were incubated at 65°C for 3 min and allowed to reanneal by incubating at 4°C for 4 h.

### RNA electrophoretic mobility shift assays

RNA electrophoretic mobility shift assays (RNA-EMSA) were performed as described previously (45). In brief, varying concentrations of purified BosR-(His) 6 protein (from 0 to 1000 nM) were incubated with 50 nM renatured, radiolabeled *rpoS* RNA probes of varying sizes in a binding buffer containing 10 mM NaH_2_PO_4_, 1 mM EDTA, 30 mM NaCl, 0.1 mM DTT and 5% glycerol. The reaction mixture was incubated at room temperature for 30 mins. Loading dye (6x Promega) was added and samples were run on temperature equilibrated 8–10% native acrylamide gels for 3 h at 50 V in ice. Dried gels were exposed to X-ray film and developed by conventional methods.

## Results

### The putative DNA binding sites of BosR are not required for the activation of the *rpoS* promoter

The regulation of RNA levels is a complex process involving not only the transcriptional control of gene expression but also post-transcriptional mechanisms such as RNA stability and degradation (46). Despite observations that *rpoS* mRNA levels decrease upon inactivation of *bosR* (16,31,33), there is still a lack of *in vivo* evidence supporting the notion that BosR regulates the transcriptional activation of *rpoS*. To investigate whether BosR regulates *rpoS* mRNA at the transcriptional or post-transcriptional level, a reporter system BS1^+^/BS2^+^*-luc* was developed by fusing a luciferase (*luc*) ORF with 250 bp upstream of the *rpoS* ORF containing the sigma54-type *rpoS* promoter and the BosR binding sites BS1 and BS2 identified *in vitro* previously (16,33,47) (Supplementary Figure S1A). To validate whether this reporter faithfully reflects native *rpoS* transcriptional regulation, the pBSV2G-based shuttle vector carrying the constructed reporter fragment was then transformed into wild-type *B. burgdorferi* strain B31, or an isogenic *rpoN* mutant, and an *rrp2^G239C^* mutant. Both native *rpoS* mRNA and *luc* mRNA levels were upregulated with elevated cell density in wild-type *B. burgdorferi* (Supplementary Figure S1B), suggesting that the constructed *luc* reporter is regulated similarly to the native *rpoS* gene. Furthermore, both native *rpoS* mRNA and *luc* mRNA levels were dramatically reduced in the *rpoN* or *rrp2^G239C^* mutant (Supplementary Figure S1C), indicating that *luc* expression is dependent on RpoN and Rrp2. These results indicate that the *luc* reporter expression faithfully represents the *rpoS* transcription activity from the –24/–12 promoter.

**Figure 1.**
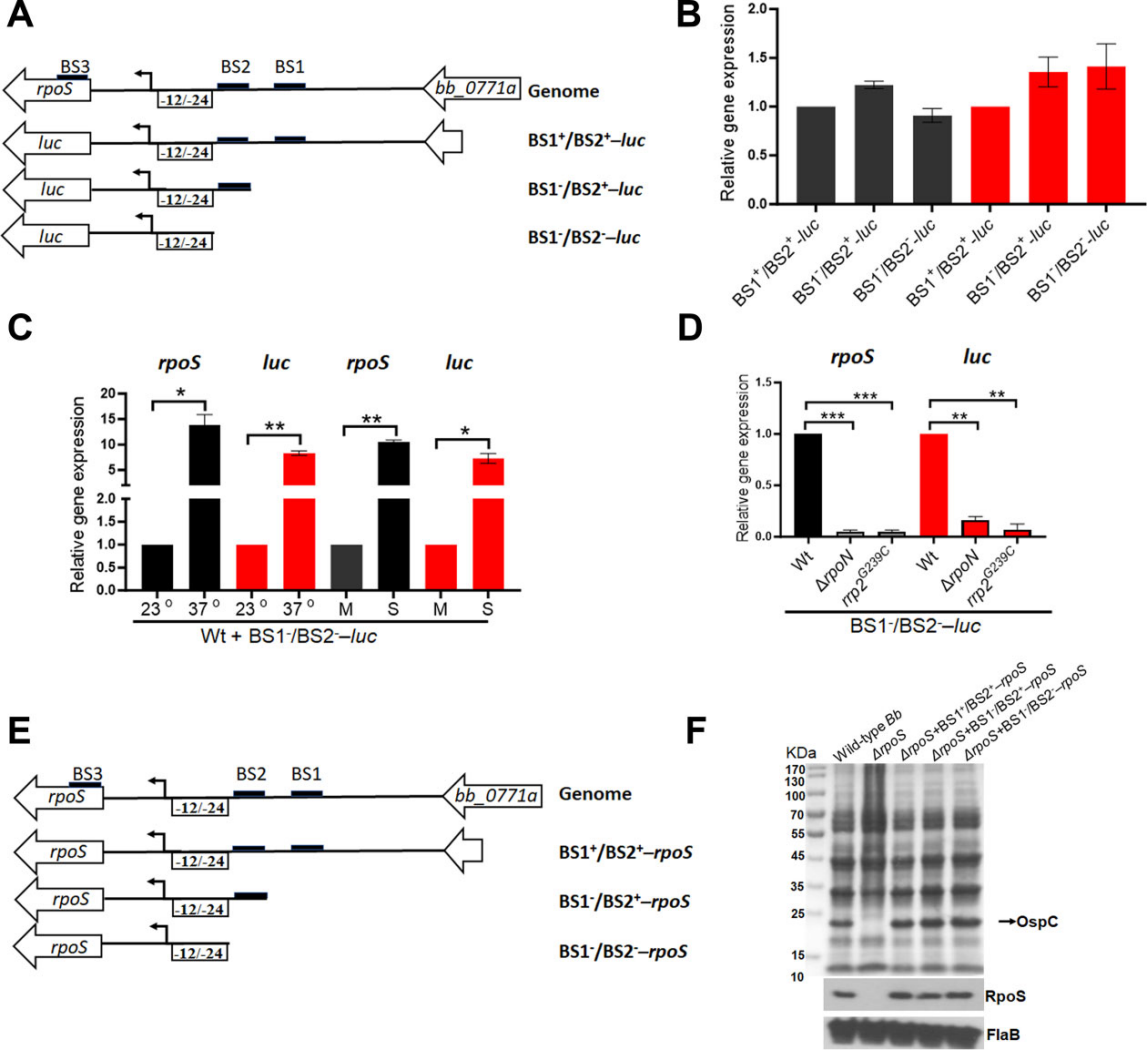
Effects of BS1 and BS2 on the *rpoS* promoter activity. (**A**) Schematic representation of the reporter constructs. The top diagram illustrates the organization of *rpoS* gene in the genome. The lower diagrams depict the *luc* gene fused with a full-length *rpoS* promoter containing both BS1 and BS2 and 5′UTR*_rpoS_* (BS1^+^/BS2^+^-*luc*), a *rpoS* promoter with only BS2 and 5′UTR*_rpoS_* (BS1^−^/BS2^+^-*luc*), or solely the sigma54-type minimal promoter and 5′UTR*_rpoS_* (BS1^−^/BS2^−^-*luc*), respectively. (**B**) Effect of BS1 and BS2 on *luc* transcript levels. Wild-type *B. burgdorferi* strains harboring each reporter plasmid were cultured in BSK-II medium at 37^o^C and harvested at the stationary phase. RNAs were extracted and subjected to qRT-PCR analyses for expressions of *rpoS* and *luc*. The levels of *rpoS* and *luc* expression in the strain containing BS1^+^/BS2^+^-*luc* were normalized to 1.0. **(C)** Effects of temperature and cell density on *luc* expression under the control of the minimal *rpoS* promoter (BS1^−^/BS2^−^-*luc*). Wild-type *B. burgdorferi* strain B31 (Wt) carrying BS1^−^/BS2^−^-*luc* was cultured in BSK-II medium either at 23°C and 37°C and harvested at mid-log (M) or stationary (S) phases. RNAs were extracted and subjected to qRT-PCR analyses. The expression levels of both *rpoS* and *luc* isolated from 23°C and mid-log culture were set as 1.0. (**D**) Dependency of the expression of BS1^−^/BS2^−^-*luc* on Rrp2 and RpoN. Various strains carrying BS1^−^/BS2^−^-*luc* were cultured at 37°C and harvested at stationary phase and RNAs were subjected to qRT-PCR. The levels of *rpoS* and *luc* transcripts in wild-type *B. burgdorferi* were set as 1.0. All bars represent the mean values of three independent experiments, and the error bars represent the standard deviation. **P< 0.001, *P < 0.01 and ***P< 0.0001 respectively using one-way ANOVA. (**E**) Schematic representation of *rpoS* complementation constructs. The top diagram illustrates the organization of the *rpoS* gene in the genome. The lower diagrams depict the *rpoS* gene along with a full-length *rpoS* promoter containing both BS1 and BS2 and 5′UTR*_rpoS_* (BS1^+^/BS2^+^-*rpoS*), a *rpoS* promoter with only BS2 and 5′UTR*_rpoS_* (BS1^−^/BS2^+^-*rpoS*), or solely the sigma54-type minimal promoter and 5′UTR*_rpoS_* (BS1^−^/BS2^−^-*rpoS*), respectively. (**F**) Effect of BS1 and BS2 on RpoS production. Wild-type *B. burgdorferi* strain 5A14 and the *rpoS* mutant (Δ*rpoS*) carrying a shuttle plasmid of BS1^+^/BS2^+^-*rpoS*, BS1^−^/BS2^+^-*rpoS*, or BS1^−^/BS2^−^-*rpoS*, were cultured in BSK-II medium at 37°C and harvested at the stationary phase. Cell lysates were then subjected to SDS analysis (top panel) or immune blot analysis (bottom panel) using antibodies against RpoS and FlaB (loading control).

To determine whether BS1 and BS2 play a role in the transcriptional activation of *rpoS*, two additional luciferase reporters were constructed, one lacking BS1 (BS1^−^/BS2^+^*-luc*) and the other lacking both BS1 and BS2 (BS1^−^/BS2^−^*-luc*) (Figure 1A). The levels of *luc* mRNA were compared among *Borrelia* strains carrying each of these constructs. The result showed that deletion of BS1 or both BS1 and BS2 did not cause any significant change in *luc* mRNA levels (Figure 1B), suggesting that BS1 and BS2 are not required for the transcriptional activation of *rpoS* at its –24/–12 promoter. This *luc* expression under the control of the minimal –24/–12 *rpoS* promoter lacking BS1 and BS2 remained regulated by culture temperature and cell density (Figure 1C) and remained dependent on RpoN and Rrp2 (Figure 1D).

To gather additional evidence, the *rpoS* deletion mutant was complemented with a native *rpoS* gene containing a 250 bp upstream region (BS1^+^/BS2^+^–*rpoS*, including the –24/–12 *rpoS* promoter and the BosR binding sites BS1 and BS2), a 95 bp upstream region (BS1^−^/BS2^+^–*rpoS*, including the –24–12 *rpoS* promoter and the BS2 sequence), or a 78 bp upstream region (BS1^−^/BS2^−^–*rpoS*, including the –24–12 *rpoS* promoter only), respectively (Figure 1E). The RpoS protein level from BS1^−^/BS2^−^–*rpoS* remained identical when compared to the level from BS1^+^/BS2^+^–*rpoS* or BS1^−^/BS2^+^–*rpoS* (Figure 1F), further supporting the notion that BS1 and BS2 are dispensable for the activation of the *rpoS* promoter.

### BosR is not required for the activation of the *rpoS* promoter

**Figure 2.**
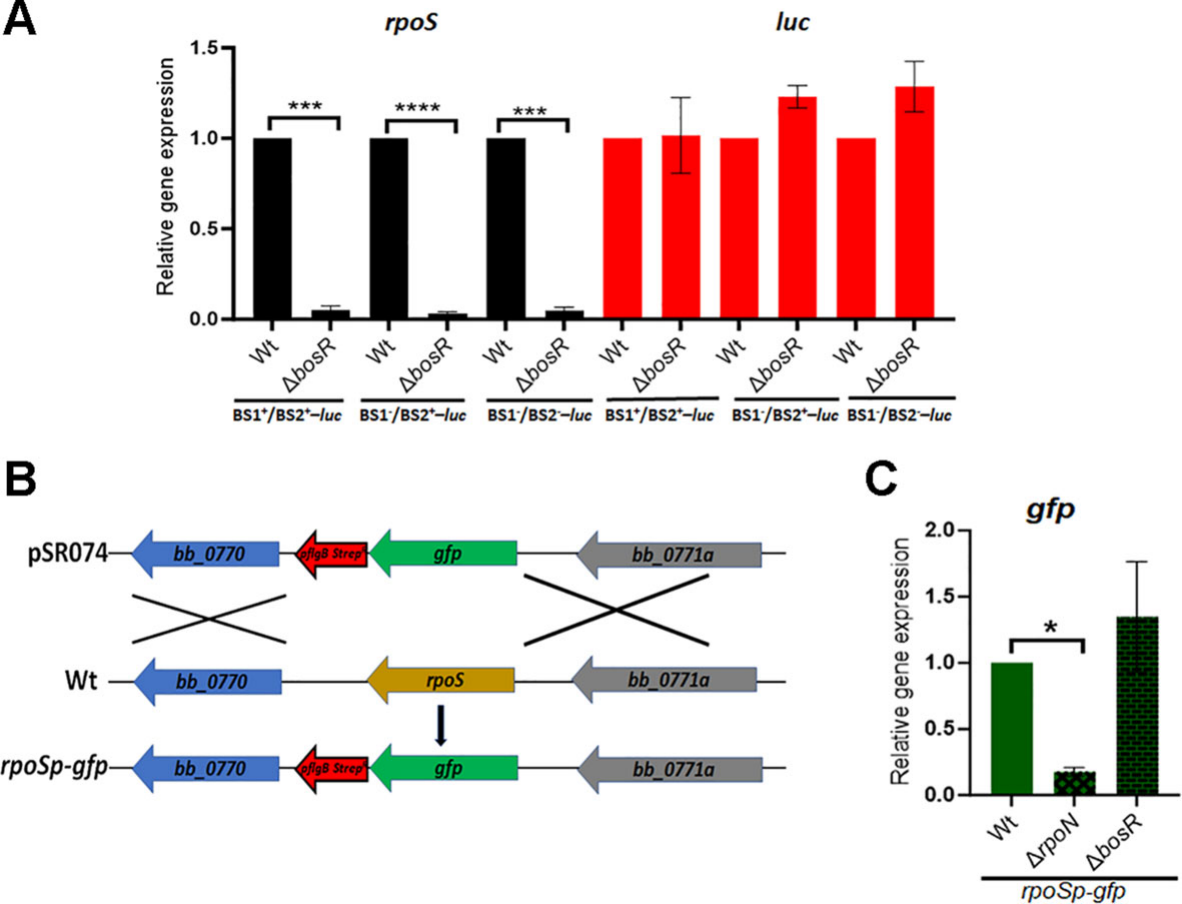
BosR does not control the *rpoS* promoter activity. (**A**) qRT-PCR analyses of the *luc* mRNA levels. Wild-type *B. burgdorferi* (Wt) strain B31 or the *bosR* mutant (Δ*bosR*) carrying a shuttle vector harboring various *luc* reporters, was cultured in BSK-II medium at 37^o^C and harvested at the stationary phase. RNAs were extracted and subjected to qRT-PCR quantitation of *rpoS* and *luc* mRNA levels. The levels of *rpoS* and *luc* transcripts in the wild-type strain were normalized to 1.0. The bars represent the mean values of three independent experiments, and the error bars represent the standard deviation. ****P < 0.00001, ***P < 0.0001, respectively using one-way ANOVA. (**B**) Strategy for replacing the native *rpoS* with a *gfp* reporter gene in the chromosome. pSR074 is a suicidal vector used for chromosomal replacement of the *rpoS* ORF with a codon optimized *gfp* ORF (68). (**C**) qRT-PCR analyses of the *gfp* mRNA levels in various strains whose native *rpoS* gene was replaced with *gfp* (*rpoSp-gfp*). Spirochetes were cultured in BSK-II medium at 37°C and were harvested at the stationary phase. RNAs were extracted and subjected to qRT-PCR analyses for the expression level of *gfp*. The expression level of *gfp* in wild-type *B. burgdorferi* with *rpoS* replaced by *gfp* was set as 1.0. The bars represent the mean values of three independent experiments, and the error bars represent the standard deviation. *P < 0.01 using one-way ANOVA.

To determine whether BosR plays a role in the transcriptional activation of *rpoS*, the luciferase reporter shuttle vectors described above were transformed into the *bosR* deletion mutant, and both native *rpoS* mRNA and *luc* mRNA levels were measured. Consistent with previous findings (29,31,33), the *rpoS* mRNA levels were significantly decreased in all reporter strains lacking BosR when compared to wild-type strains (Figure 2A). However, no difference was observed in *luc* mRNA levels between wild-type strains and the *bosR* mutant strains (Figure 2A). These findings suggest that while BosR is required for achieving a high level of *rpoS* mRNA, it does not play a role in the transcriptional activation of *rpoS*. In other words, BosR regulates *rpoS* mRNA at the post-transcriptional level, not at the transcriptional level.

To further strengthen this finding, chromosomal *rpoS* promoter-tagged *gfp* reporters (*rpoSp-gfp*) were constructed by substituting the *rpoS* ORF with a *gfp* ORF, in the wild-type, the *rpoN* mutant, or the *bosR* mutant, respectively (Figure 2B). The result showed that while the *rpoN* mutant exhibited a dramatic reduction in *gfp* transcript levels, there was no significant difference in *gfp* transcript levels between wild-type *B. burgdorferi* and the *bosR* mutant (Figure 2C), further supporting the notion that BosR is not required for the transcriptional activation of *rpoS*.

### The level of artificially transcribed *rpoS* mRNA is dependent on BosR

**Figure 3.**
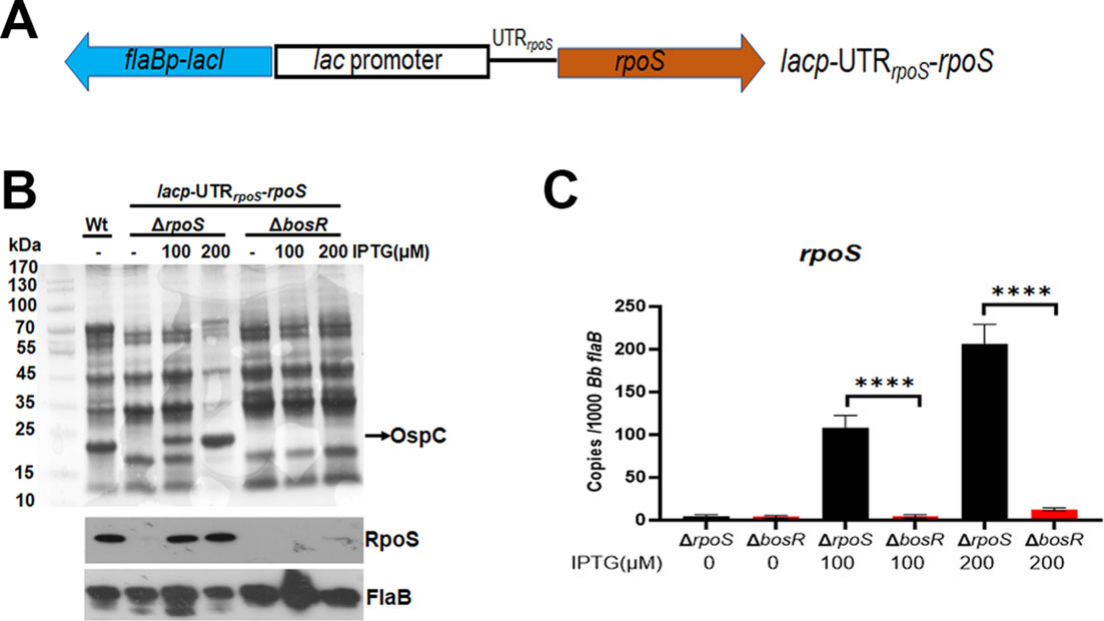
Artificial induction of *rpoS* expression in the *bosR* mutant. (**A**) Schematic representation of the shuttle vector carrying an IPTG-inducible *rpoS* gene (*lacp*-UTR*_rpoS_*-*rpoS*). The blue arrow labeled *flaBp*-*lacI* represents a *flaB* promoter-driven *lacI* gene. The *lac* promoter element, depicted in a square box (including the –35/–10 promoter and the operator), is fused with a fragment containing UTR*_rpoS_* and *rpoS* ORF (brown arrow). (**B**) Coomassie gel staining and Immunoblotting of OspC and RpoS levels. The *rpoS* mutant (Δ*rpoS*) or *bosR* mutant (Δ*bosR*), containing the *lacp*-UTR_rpoS_-*rpoS* plasmid, along with a wild-type *B. burgdorferi* strain AH130 as a control, were cultured in BSK-II medium with various concentrations of IPTG (indicated on top) for 4 days (with an initial concentration of 1 × 10^4^ spirochetes/ml) and then subjected to SDS analysis (top panel) or immunoblotting (bottom panel) using antibodies against RpoS and FlaB (loading control). (**C**) qRT-PCR analyses of *rpoS* mRNA levels. RNAs were extracted from the same cultures as above and were subjected to qRT-PCR analyses. The values represent the *rpoS* mRNA copies normalized to 1000 copies of *B. burgdorferi flaB* mRNA. The bars represent the mean values of three independent experiments, and the error bars represent the standard deviation. ****P < 0.00001 using one-way ANOVA.

To gain further evidence that BosR does not regulate *rpoS* mRNA at the level of transcription, a shuttle vector harboring an IPTG-inducible *rpoS* ORF along with a 50 bp 5′ UTR (*lacp*-UTR*_rpoS_*-*rpoS*) was transformed into either the *rpoS* or *bosR* mutant, respectively (Figure 3A). As expected, the addition of IPTG in the *rpoS* mutant carrying *lacp*-UTR*_rpoS_*-*rpoS* resulted in an increased production of RpoS protein in an IPTG dose-dependent manner. In contrast, low or no RpoS protein was detected in the *bosR* mutant in the presence of IPTG (Figure 3B). Consistent with the RpoS protein levels, very low levels of *rpoS* mRNA were detected in the *bosR* mutant despite increased levels of IPTG (Figure 3C). At 100 μM of IPTG concentration, the *rpoS* mutant displayed a significant growth defect (Supplementary Figure S2A), consistent with the fact that overproduction of RpoS is lethal to borrelial growth (48). On the other hand, no growth defect was observed in the *bosR* mutant even at 200 μM of IPTG concentration (Supplementary Figure S2B), consistent with the lower levels of RpoS in these strains. These genetic data further strengthen the conclusion that BosR regulates *rpoS* mRNA post-transcriptionally.

### BosR controls the turnover rate of *rpoS* mRNA

To investigate how BosR regulates *rpoS* mRNA post-transcriptionally, we first determined the effect of BosR on the turnover rate of *rpoS* mRNA, as controlling mRNA turnover rate is a common mechanism for post-transcriptional regulation in bacteria (49). Accordingly, we compared the turnover rates of *rpoS* mRNA between wild-type *B. burgdorferi* and the *bosR* mutant. Since *B. burgdorferi* is naturally resistant to rifampin, a commonly used antibiotic for transcription arrest, we used actinomycin D as a transcription inhibitor to measure *rpoS* mRNA decay(41,50–52). After actinomycin-D treatment, the decay profiles of *rpoS* mRNA were compared across the strains. As a control, the decay kinetics of *flaB* mRNA in both strains were also quantified.

**Figure 4.**
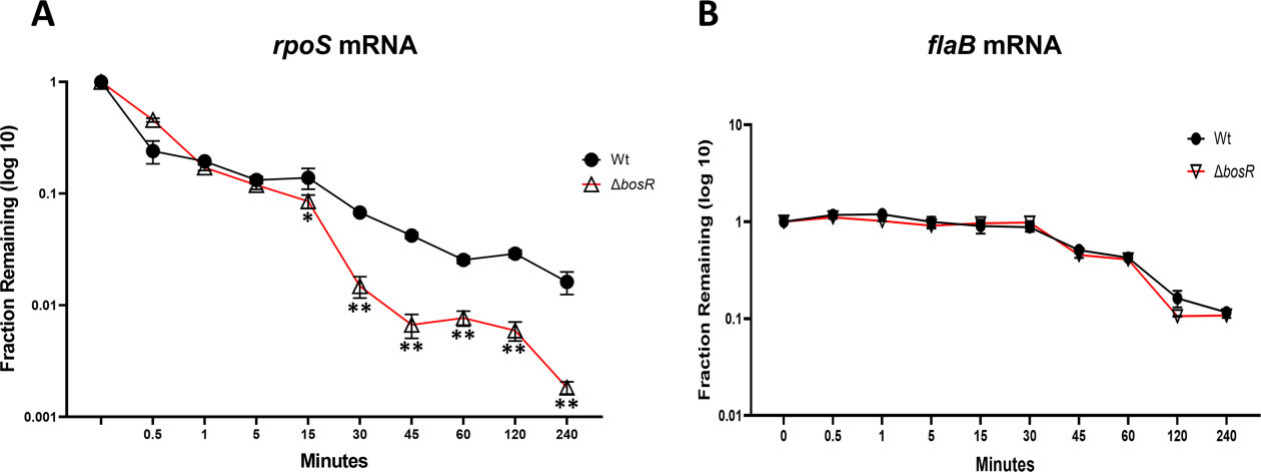
*rpoS* mRNA decay curves across wild type and *bosR* mutant. Wild-type *B. burgdorferi* strain B31 (Wt) and the *bosR* mutant (Δ*bosR*) were cultured in BSK-II at 37°C to stationary phase. Transcriptional arrest was induced by adding actinomycin D (150 μg/ml) (41), and samples were collected at various time points after the actinomycin D treatment. RNAs were extracted and subjected to qRT-PCR analyses for quantification of the copy numbers of *rpoS* mRNA (**A**) or *flaB* mRNA (**B**). The fraction of remaining RNA (*f*) was calculated and plotted as log values. Closed circles and open triangles represent the remaining RNA fraction in wild-type *B. burgdorferi* and the *bosR* mutant, respectively. The error bars represent standard deviation of three independent experiments. * *P* < 0.01; ** *P* < 0.001, using *t*-test.

Consistent with previous reports (41), multi-phased mRNA decay for *rpoS* transcripts was observed in both strains. The first decay phase occurred within 1 min after actinomycin-D treatment. The principal consequence of the *bosR* deletion was observed in the alteration of decay kinetics following the initial phase. The fraction of *rpoS* RNA remaining in the *bosR* mutant after 45 minutes was about tenfold lower compared to that in the wild-type strain (Figure 4A). The decay rate of *flaB* mRNA was not affected by the *bosR* mutant (Figure 4B), suggesting that BosR affecting the decay rate of *rpoS* mRNA is specific.

### BosR binds *rpoS* mRNA *in cellulo*

**Figure 5.**
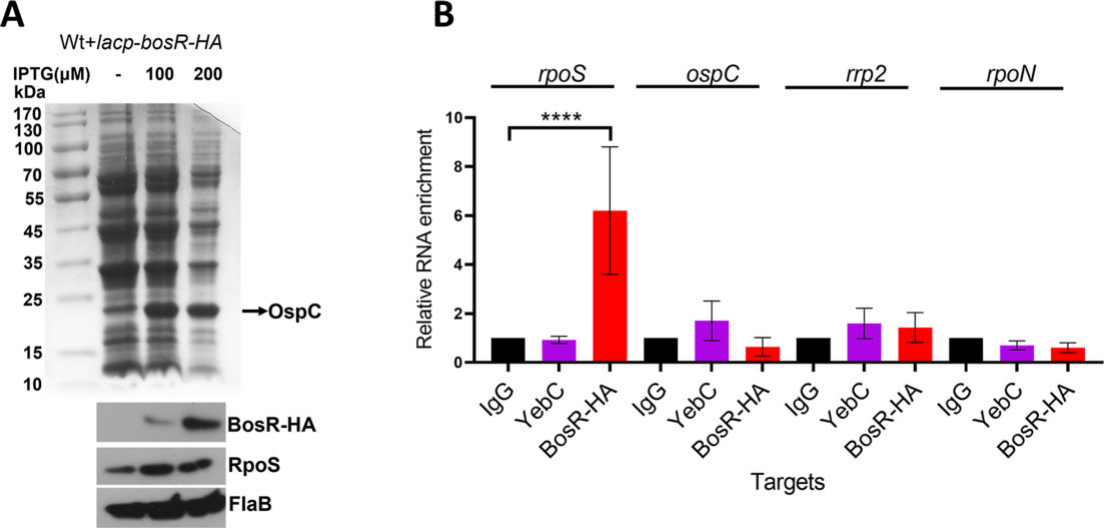
RNA immunoprecipitation (RIP) demonstrates BosR binding to *rpoS* mRNA *in cellulo*. (**A**) IPTG induction of HA-tagged BosR. Wild-type *B. burgdorferi* strain B31 expressing a shuttle vector carrying *lacp-bosR-HA* was cultured in BSK-II at 37°C in the presence of various concentrations of IPTG. Spirochetes were harvested at stationary phase and subjected to SDS-PAGE (top panel) and immunoblotting analyses (lower panel). (**B**) RNA immunoprecipitation. Spirochetes grown in the presence of 100 μg/ml of IPTG were subjected to RIP using anti-HA, anti-YebC, or normal mouse IgG. Immunoprecipitated RNA samples were subjected to qRT-PCR analyses to determine the copy numbers of *rpoS, ospC*, *rrp2*, and *rpoN* mRNAs (labeled on top). The mRNA levels for each gene in BosR-HA and YebC samples were normalized with the mRNA levels of the IgG sample for each gene (in which the value was set as 1.0). The bars represent the mean values of three independent experiments, and the error bars represent the standard deviation. ****P < 0.00001, using one-way ANOVA.

To investigate whether BosR regulates the turnover rate of *rpoS* mRNA by binding to *rpoS* mRNA *in cellulo*, we performed RNA immunoprecipitation assays using a *B. burgdorferi* strain carrying a shuttle vector harboring an IPTG-inducible, modified *bosR* gene that encodes a BosR-HA fusion protein (*lacp-bosR-HA*). The resulting strain was cultured in the presence or absence of IPTG. Production of BosR-HA fusion protein upon addition of IPTG was confirmed by immunoblotting (Figure 5A). Cell lysates were subjected to immunoprecipitation using anti-HA for BosR-HA, using the antibody for YebC, another transcriptional regulator that regulates *vlsE* expression (42), or using anti-mouse IgG for nonspecific binding control. The presence of BosR-HA proteins in the anti-HA antibody-immunoprecipitated sample was confirmed by immunoblotting (Supplementary Figure S3). The immunoprecipitated samples were then subjected to RNA extraction and qRT-PCR analyses. The result showed that *rpoS* mRNA was readily detected in the BosR-HA immunoprecipitated samples, but not in the samples using anti-YebC antibody or IgG (Figure 5B). These findings suggest that BosR binds *rpoS* mRNA in the cell.

### BosR binds to the 5′ UTR region of *rpoS* mRNA

**Figure 6.**
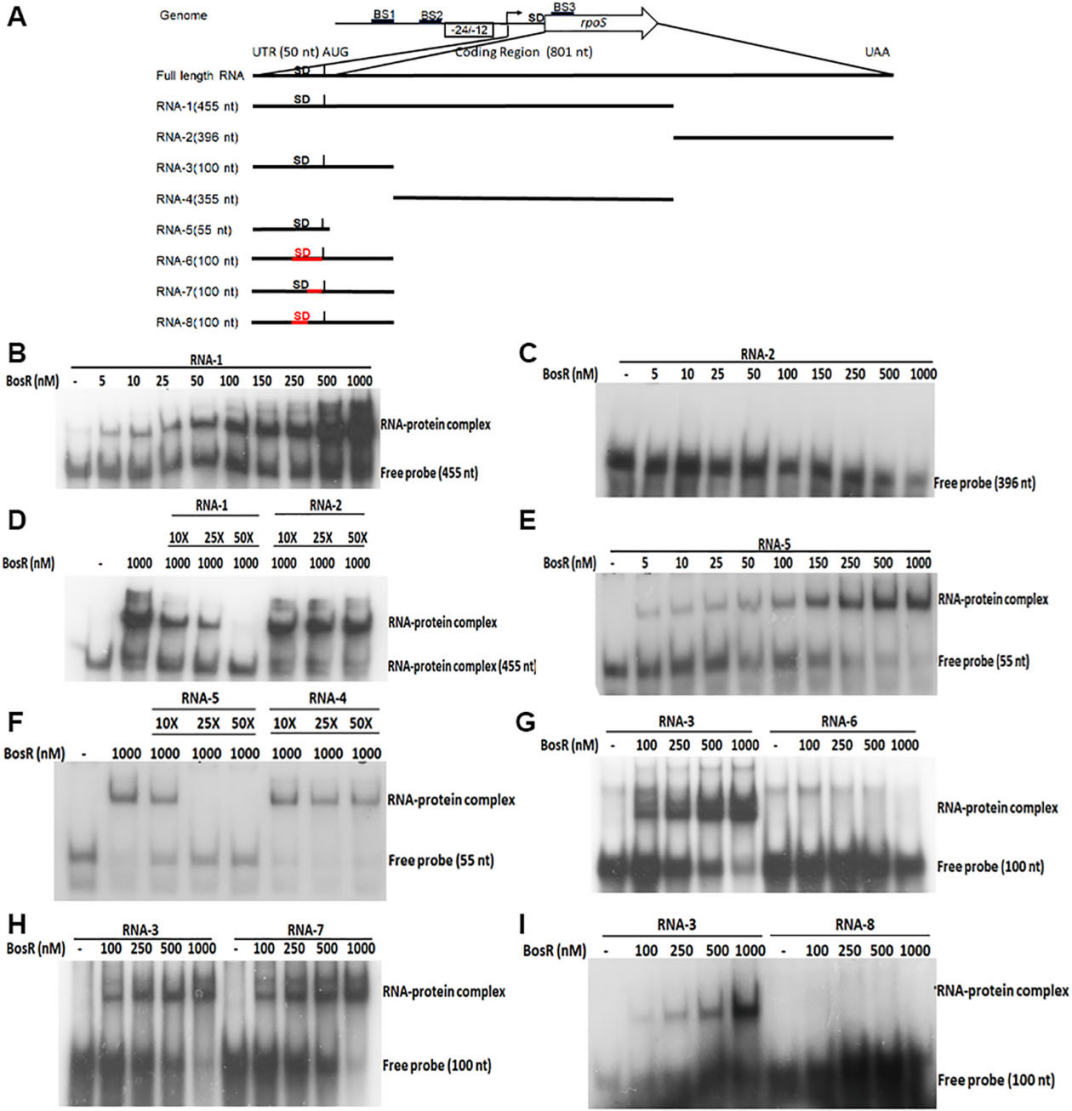
Identification of BosR binding region in *rpoS* mRNA using RNA electrophoretic mobility shift assay (EMSA). (**A**) Schematic representation of RNA species used for RNA EMSA. The horizontal line represents the respective RNA species used for the study. The length of each RNA species is represented at the end of each line. The vertical line indicates the AUG sequence, and the 5′UTR region (50 nt long) is labeled on top. Shine-Dalgarno sequence is labeled as SD. Red line indicates the mutagenized regions within the 5′UTR sequence. (**B–I**) RNA EMSA using BosR protein and various lengths of *rpoS* mRNA. For all the EMSA, 50 nM of each RNA species were incubated with varying concentrations of BosR (indicated on top). ^32^P-labeled or non-labeled RNAs were produced by *in vitro* transcription. The RNA-protein complex and free probes with respective sizes are indicated on the right of each figure. For competitive EMSA, 10 to 50 folds of cold RNAs were used (labeled on top).

To map the BosR binding region within *rpoS* mRNA, *in vitro* RNA electrophoretic mobility shift assays (EMSA) were performed using recombinant BosR and various lengths of *in vitro* transcribed ^32^P-labeled *rpoS* mRNAs (Figure 6A). The result showed that recombinant BosR could bind to 455 nt of the 5′ *rpoS* RNA (RNA-1) (Figure 6B), even at 5 nM concentration of BosR. However, no BosR binding was observed with the remaining 396 nt of the 3′ RNA probe (RNA-2) (Figure 6C). BosR binding to the 5′ part of *rpoS* mRNA was specific, as the competitive EMSA showed that an excess amount of non-labeled RNA-1 could compete off the binding, while RNA-2 could not (Figure 6D).

To further identify the BosR binding region within the 5′ region of *rpoS* mRNA, RNA-1 was divided into two fragments: 100 nt of 5′ fragment (RNA-3) and 3′ fragment of 355 nt (RNA-4). The result showed that BosR binds to RNA-3, not RNA-4 (Figure 6F, G). We further found that BosR binds to the 5′ fragment of 55 nt of RNA-3, named RNA-5 (Figure 6E). RNA-5 contains 50 nt of 5′ UTR sequence of *rpoS* mRNA plus 5 nt of the *rpoS* coding sequence. BosR binding to RNA-5 was specific, demonstrated by the competitive EMSA assays (Figure 6F). The dissociation constant (*K*_D_) of BosR binding to RNA-5, calculated from three independent experiments, was 25 nM ± 0.5 (Supplemental Figures S4). To further narrow down which region of the 5′UTR sequence is involved in BosR binding, parts of the sequence were replaced with corresponding 5′UTR sequences of *flaB* (RNA-6, RNA-7, RNA-8, Figure 6G–I). The result showed that replacing the 10 nt sequence at position 30–40 of *rpoS* 5′UTR with the corresponding *flaB* sequence led to a loss of BosR binding (Figure 6I). Taken together, these results demonstrate that BosR directly binds to *rpoS* mRNA, and the region at positions 30–40 within 5′ UTR of *rpoS* mRNA is important for BosR binding.

### BosR exhibits a higher binding affinity for *rpoS* mRNA than for its DNA target

**Figure 7.**
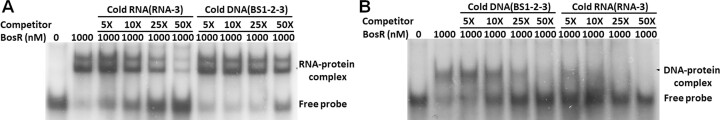
BosR has higher affinity towards *rpoS* mRNA than *rpoS* promoter DNA. (**A**) BosR-*rpoS* mRNA (RNA-3) binding was competed with 5- to 50-fold excess of cold RNA-3 or 560 bp cold DNA fragments containing BS1, BS2 and BS3 sequences. (**B**) BosR-DNA binding of the *rpoS* promoter (560 bp containing BS1, BS2 and BS3 sequence) was competed with 5- to 50-fold excess of cold DNA fragments or cold RNA-3.

Previously, BosR was shown capable of binding to DNA *in vitro* (16,33,53). Since the result above demonstrates BosR as an RNA-binding protein, we thought to compare the affinity of BosR towards *rpoS* mRNA and *rpoS* promoter DNA. Accordingly, BosR-mRNA (RNA-3) binding was outcompeted with an excess amount of either cold RNA-3 or cold DNA fragment (560 bp containing BS1, BS2, and BS3 sequences, respectively). The result showed that, unlike cold RNA-3 probes, the cold DNA probe was not able to compete off the binding even in the presence of a 25-fold excess of cold DNA probe (Figure 7A). Competition of BosR-DNA binding with cold RNA was also performed (Figure 7B). Both cold DNA and RNA probes were able to compete off BosR-DNA binding, but cold RNA probes competed more efficiently than cold DNA probes (Figure 7B). This result suggests that BosR has a stronger affinity for *rpoS* mRNA than the previously identified DNA target.

### The 5′UTR region is required for *rpoS* mRNA degradation

**Figure 8.**
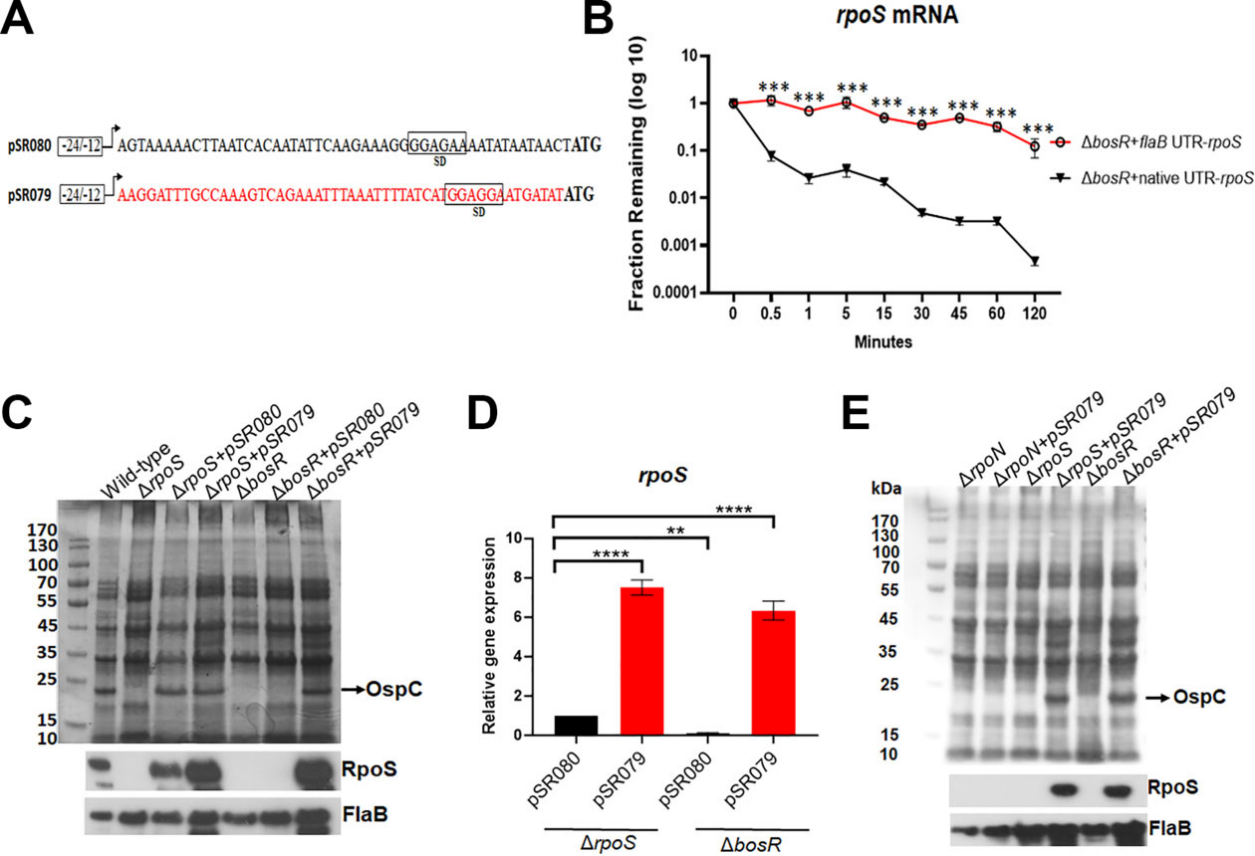
The role of 5′ UTR on *rpoS* expression. (**A**) Schematic representation showing the mutated 5′ UTR *rpoS* gene on the shuttle vector. The open box labeled with –24–12 represents the major *rpoS* promoter (a σ^54^-type promoter). The arrow indicates the transcription start site (TSS) of *rpoS*. Shine-Dalgarno sequence is represented in a square box labeled as SD. The ATG start codon of *rpoS* ORF is in bold. pSR080 harbors a native copy of *rpoS* with its minimal σ^54^-type promoter. pSR079 harbors a mutated version of *rpoS* in which the 5′UTR was replaced with a 50bp *flaB* UTR (indicated in Red). (**B**) RNA decay assays. The *bosR* mutant carrying either pSR079 or pSR080 was subjected to RNA decay assays as described in Figure 5. Open circles and closed triangles represent the remaining RNA fraction. The error bars represent the standard deviation of three independent experiments. ***P < 0.0001, using *t*-test. (**C**) The effect of 5′ UTR on RpoS production. Wild-type *B. burgdorferi* 5A14, the *rpoS* mutant (Δ*rpoS*), the *rpoS* mutant containing pSR080 (Δ*rpoS +*pSR080) or pSR079 (Δ*rpoS +*pSR079), and the *bosR* mutant (Δ*bosR*), the *bosR* mutant containing pSR080 (Δ*bosR +*pSR080) or pSR079 (Δ*bosR +*pSR079), were cultured in BSK-II medium at 37°C and harvested at the stationary phase. Cell lysates were subjected to SDS-PAGE (top panel) or immunoblot analyses (bottom panel). The bands corresponding to OspC, RpoS and FlaB were indicated on the right. (**D**) Quantitation of *rpoS* mRNA levels by qRT-PCR. RNAs were extracted from the cultures in (C) and subjected to qRT-PCR. The levels of *rpoS* mRNA in Δ*rpoS +*pSR080 were normalized as 1.0. The bars represent the mean values of three independent experiments, and the error bars represent the standard deviation. ****P < 0.00001; **P < 0.001 using one-way ANOVA. (**E**) BosR-independent *rpoS* expression from pSR079 requires RpoN. Spirochetes were cultured as described in (C) and cell lysates were subjected to SDS-PAGE (top panel) or immunoblot analyses (bottom panel). Bands corresponding to RpoS, OspC and FlaB are indicated on the right.

Given that BosR binds to the 5′UTR of *rpoS* mRNA, we sought to investigate whether the 5′UTR plays a role in *rpoS* mRNA stability. Accordingly, two shuttle vectors were constructed: one carrying a wild-type copy of *rpoS* with its 5′ UTR sequence (5′UTR*_rpoS_*-*rpoS*, pSR080), and the other carrying an *rpoS* gene but its entire 50 bp 5′UTR was replaced with the 50 bp 5′ UTR sequence of the *flaB* gene (5′UTR*_flaB_*-*rpoS*, pSR079). The generated constructs were transformed into the *rpoS* or *bosR* mutant, respectively (Figure 8A). We first confirmed that BosR failed to bind to the mutated *rpoS* mRNA containing the *flaB* 5′UTR sequence (Supplementary Figure S5A). We then analyzed the turnover rate of 5′UTR*_rpoS_*-*rpoS* RNA and 5′UTR*_flaB_*-*rpoS* mRNA in the *bosR* mutant. As expected, 5′UTR*_rpoS_*-*rpoS* mRNA showed a similar rapid decay profile as the native *rpoS* mRNA in the *bosR* mutant (Figures 4 and 8B). Strikingly, 5′UTR*_flaB_*-*rpoS* RNA showed a much more stable profile (Figure 8B), with a decay rate similar to what was observed for *flaB* mRNA (Figure 4). This result suggests that the 5′ UTR region is responsible for the quick turnover rate of *rpoS* mRNA.

### The 5′UTR region requires BosR to prevent degradation

To investigate whether the mutated *rpoS* mRNA containing the *flaB* 5′UTR still requires BosR for stability, shuttle vectors pSR079 or pSR080 were transformed into the *rpoS* and *bosR* mutants. Both plasmids were able to complement RpoS and OspC production in the *rpoS* mutant (Figure 8C), except that pSR079 (5′UTR*_flaB_*-*rpoS*) showed a higher RpoS level than pSR080 (5′UTR*_rpoS_*-*rpoS*). As expected, the *bosR* mutant carrying pSR080 failed to produce RpoS and OspC. Importantly, the *bosR* mutant carrying pSR079 showed high levels of RpoS production (Figure 8C). Consistent with what was observed at the protein level, both *rpoS* and *bosR* mutants carrying pSR079 showed 6–7-fold higher *rpoS* mRNA levels than both mutants carrying pSR080 (Figure 8D). These results along with the observation above, suggest that *rpoS* mRNA lacking the native 5′UTR not only results in a highly stable RNA but also does not require BosR for its stability.

To determine whether this BosR-independent RpoS production from 5′UTR*_flaB_*-*rpoS* remained to be RpoN-dependent, pSR079 and pSR080 were transformed into the *rpoN* deletion mutant. No RpoS was detected in the *rpoN* mutant carrying either pSR079 or pSR080 (Figure 8E). This result confirms that 5′UTR*_flaB_*-*rpoS* mRNA was transcribed from the σ^54^-type promoter, which requires RpoN for transcriptional activation.

**Figure 9.**
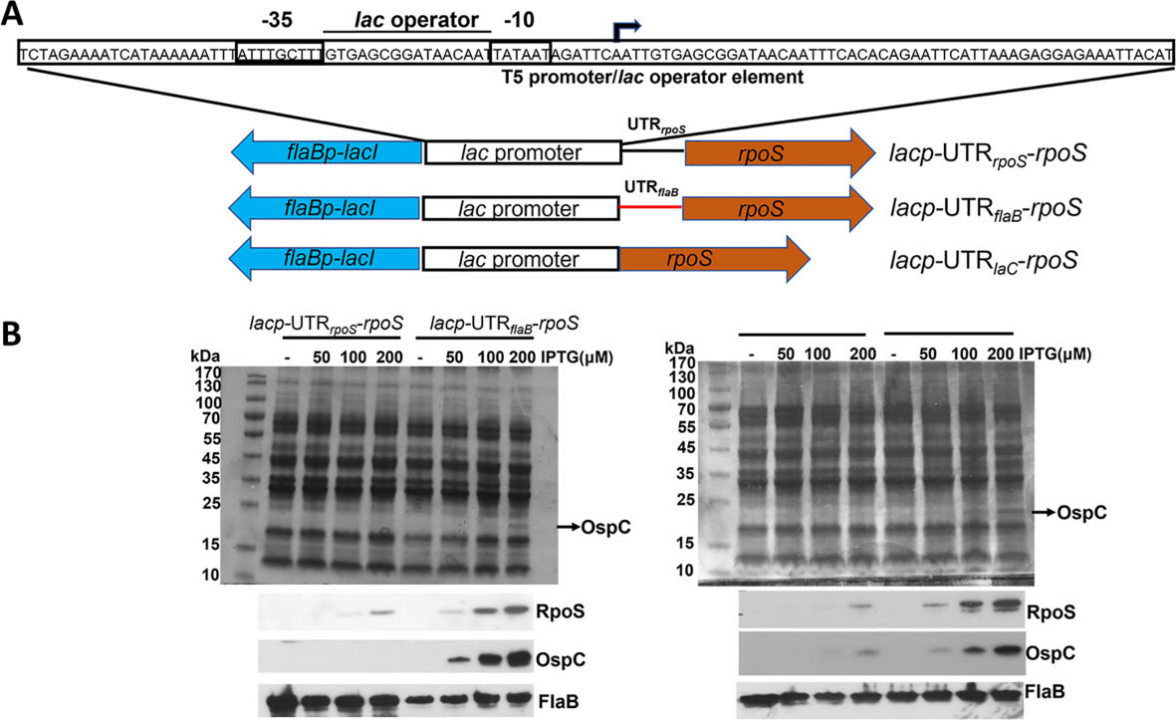
Artificial induction of altered 5′UTR versions of *rpoS* mRNA produces BosR-independent *rpoS* mRNA. (**A**) Schematic of the promoter sequences of IPTG-inducible *rpoS* on various shuttle plasmids. The blue arrow represents *flabp-lacI*, the square box represents the *lac* promoter, and the brown arrow represents the *rpoS* ORF. The 50 bp of 5′ UTR of *rpoS* or the 50 bp of of 5′ UTR of *flaB* 5′UTR is represented in black line or red line, respectively. *lacp*-UTR*_rpoS_*-*rpoS*: the shuttle plasmid carrying a *lac* promoter-driven *rpoS* with its native 5′UTR. *lacp*-UTR*_flaB_*-*rpoS*: the shuttle plasmid carrying a *lac* promoter-driven *rpoS* with a 5′UTR from the *flaB* gene. *lacp*-UTR*_laC-_rpoS*: the shuttle plasmid carrying a *lac* promoter-driven *rpoS* without 5′UTR of *rpoS*. The expanded region indicates the architecture of the *lac* (T5) promoter and operator sequences. The small open box labeled with –10 and –35 represents the *rpoD* promoter. The *lac* operator region is represented between –10 and –35 sequences. The arrow indicates the transcription start site (TSS). (**B**) Quantification of RpoS production. The *bosR* mutant (Δ*bosR*) containing either *lacp*-UTR*_rpoS_*-*rpoS* or *lacp*-UTR*_flaB_*-*rpoS* (left), or containing *lacp*-UTR*_laC_*-*rpoS* (right) was cultured in BSK-II medium with various concentrations of IPTG (indicated on top) with an initial concentration of 1 × 10^4^ spirochetes/ml and harvested at day 4. Cell lysates were then subjected to SDS-PAGE (top panel) or immunoblot analyses (bottom panel). Bands corresponding to OspC, RpoS and FlaB are highlighted on the right.

To gain further evidence that alteration of the 5′UTR of *rpoS* mRNA results in BosR-independent *rpoS* expression, the *rpoS* genes with various versions of 5′UTR were placed under the control of a *lac* promoter (Figure 9A). The result showed that consistent with what was observed in Figure 3B, no or low levels of RpoS were detected in the *bosR* mutant carrying the *rpoS* gene with its native 5′UTR (plus an additional 5′UTR sequence from the lac promoter) (Figure 9B), indicating that the presence of a native 5′UTR sequence in *rpoS* RNA requires BosR to prevent degradation. However, RpoS was readily detected from the *rpoS* gene lacking its native 5′UTR sequence, either replaced with the *flaB* 5′UTR (*lacp-*UTR*_flaB_-rpoS*) sequence or with a *lacZ* 5′UTR sequence (*lacp-*noUTR*-rpoS*) (Figure 9B). These data further support the notion that the native 5′UTR sequence of *rpoS* is required for its rapid degradation unless being protected by BosR, and lacking the *rpoS* 5′UTR sequence allows bypassing the requirement of BosR, resulting in a stabilized *rpoS* mRNA.

### The GG residues in 5′UTR are critical for both *rpoS* mRNA degradation and BosR binding

**Figure 10.**
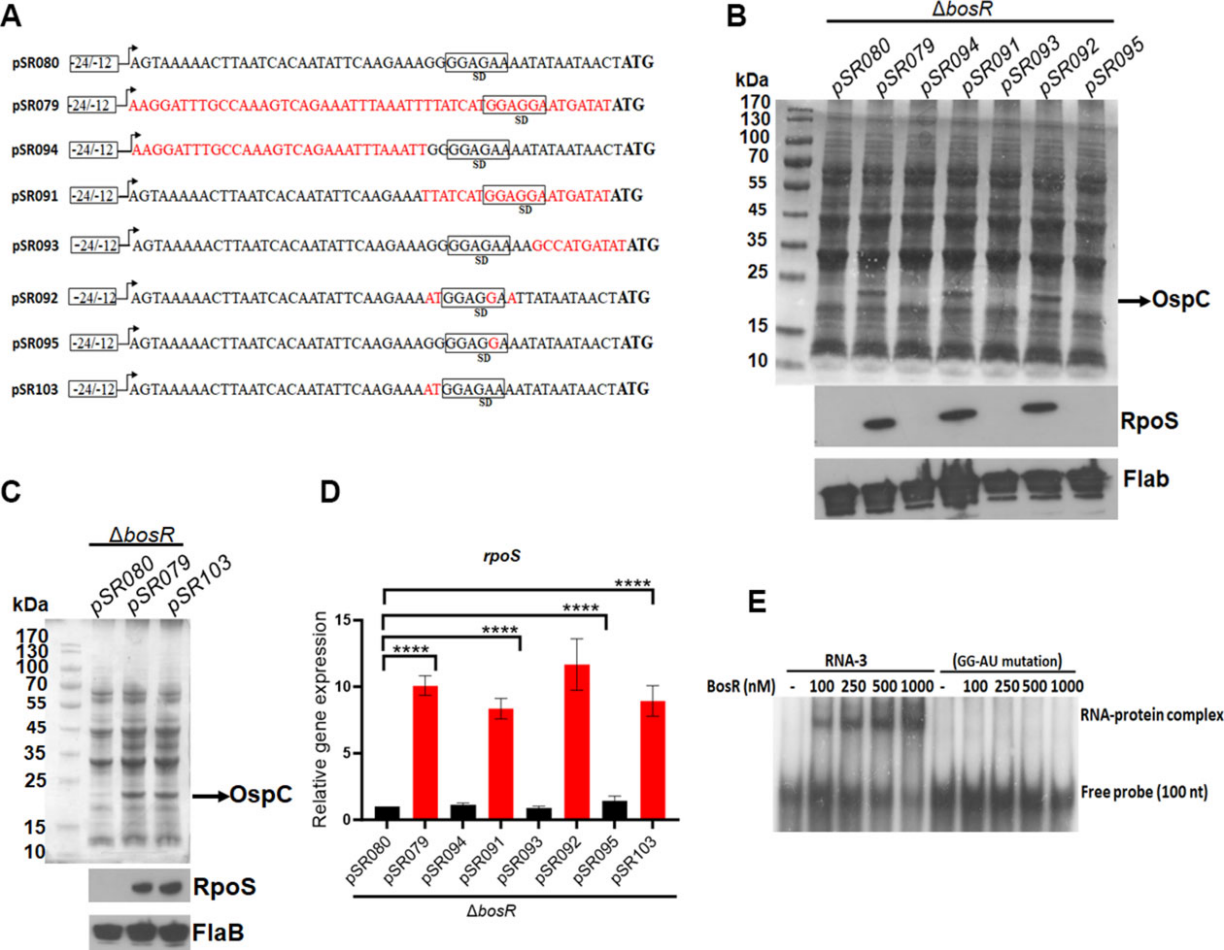
Identification of RNA nucleotides important for BosR binding and for *rpoS* mRNA stability. (**A**) Schematic representation of plasmids used for mutagenesis studies. The labels are identical to those in Figure 8A. The 50 bp sequence of the 5′ UTR of *rpoS* is depicted in black, while various mutations in the 5′ UTR are represented in red. (**B**, **C**) Effects of mutations within 5′ UTR on RpoS production. The *bosR* mutant (Δ*bosR*) carrying various shuttle vectors were cultured as described above, and RpoS and OspC productions were analyzed by SDS-PAGE and immunoblotting. (**D**) Quantitation of *rpoS* mRNA levels by qRT-PCR. Cultures of the *bosR* mutant (Δ*bosR*) carrying various shuttle vectors were subjected to RNA extraction and qRT-PCR analyses. The level of *rpoS* mRNA of the *bosR* mutant containing pSR080 (native 5′UTR sequence) was normalized to 1.0. The bars represent the mean values of three independent experiments, and the error bars represent the standard deviation. ****, *p* < 0.00001 using one-way ANOVA. (**E**) RNA EMSA with BosR protein and the *rpoS* mRNA containing GG to AU mutation. RNA-3, a 100 nt containing the native 5′UTR sequence, is described in Figure 6A. RNA-9 is RNA-3 containing GG to AU mutation at positions 30 and 31 within the 5′ UTR sequence.

The above results demonstrate the necessity of the 5′UTR sequence for BosR binding and the degradation of *rpoS* mRNA. As BosR binds to the region between residues 30–40 within the 5′UTR sequence, we sought to determine if the BosR binding site and the RNA degradation site overlap each other. Accordingly, a series of shuttle plasmids carrying the *rpoS* gene with various mutations were constructed and transformed into the *bosR* mutant (Figure 10A). When the 20-nucleotide sequence at the 3′ end of the 5′UTR sequence was replaced with the corresponding *flaB* region (pSR091), BosR-independent *rpoS* expression was observed at both the mRNA and protein levels (Figure 10B and D). Furthermore, the *bosR* mutant containing pSR092, in which the 10-nucleotide sequence at the 5′ end (positions 30–40 downstream of the transcription start site) was replaced with the corresponding *flaB* region (resulting in a 4-nucleotides difference), exhibited BosR-independent *rpoS* expression (Figure 10B and D). Given that this region is the BosR RNA-binding region identified above (Figure 6), these findings strongly suggest an overlap between the *rpoS* RNA degradation site and the BosR binding site.

The sequence from position 30–40 includes a predicted Shine–Dalgarno (SD) sequence (Figure 10A). To investigate whether the SD sequence is involved in *rpoS* RNA degradation and BosR binding, plasmid pSR095 was constructed, wherein the SD sequence of *rpoS* was converted to the SD sequence of *flaB* (pSR095). The result showed that the *bosR* mutant carrying pSR095 did not express *rpoS* RNA or protein, suggesting that the SD sequence is not crucial for rpoS mRNA degradation (Figure 10B, D). Further EMSA results indicate that BosR was still able to bind to this mutated RNA, suggesting that the SD sequence is not involved in BosR binding (Supplementary Figure S5B).

We further mutated the GG residues at positions 30 and 31 within the 5′UTR sequence (pSR103). The result showed that the *bosR* mutant carrying pSR103 expressed *rpoS* in both RNA and protein levels, suggesting that this mutated *rpoS* mRNA is stable even in the absence of BosR (Figure 10C, D). The GG mutation also completely disrupted BosR binding to *rpoS* RNA (Figure 10E). Taken together, these results suggest that the *rpoS* mRNA degradation site and the BosR binding site overlap, and the GG residues at positions 30 and 31 play a pivotal role in BosR binding to *rpoS* mRNA and in *rpoS* mRNA degradation.

## Discussion

It is well-established that in other bacteria, ATP-dependent bacterial enhancer-binding proteins (bEBP) are essential and sufficient for a σ^54^-dependent transcriptional activation *in vitro* and *in cellulo*, promoting the formation of the RNAP-σ^54^ closed complex into the open complex (54–56). The mystery of why *B. burgdorferi* EBP, Rrp2, requires another activator BosR for σ^54^-dependent activation of *rpoS* poses a significant hurdle in our understanding of the regulation of the RpoS pathway. In this study, using a promoter reporter system, we demonstrated that both BosR and the previously proposed DNA-binding sites for BosR are dispensable for σ^54^-dependent transcription activation of *rpoS in cellulo*. We further provide both genetic and biochemical evidence that BosR does not function as a transcriptional activator of *rpoS*. Instead, it regulates *rpoS* mRNA post-transcriptionally by directly binding to the 5′UTR region of *rpoS* mRNA, preventing its degradation.

One of the key observations in this study was that when *rpoS* transcription was induced by IPTG from the *lac* promoter, virtually no *rpoS* mRNA or RpoS protein was detected in the *bosR* deletion mutant (Figure 3). This result provides compelling evidence that BosR regulates the level of *rpoS* mRNA. This observation seems to contradict earlier reports showing that a *lac* promoter-driven *rpoS* mRNA was readily detected in *bosR* mutant strains in an IPTG dose-dependent manner (16,34), which strongly supports BosR’s role as a transcriptional activator of *rpoS*. However, a key difference exists in the *lac* promoter- *rpoS* fusion constructs between this study and the previous studies. In previous studies, the *lac* promoter-*rpoS* fusion constructs excluded the 5′UTR of *rpoS* mRNA sequence by directly fusing *rpoS* ORF to the *lac* promoter. In this study, the inducible *rpoS* construct included the native 5′UTR sequence of *rpoS*. We discovered that the 5′UTR region governs the fate of *rpoS* mRNA (Figures 8 and 9). Excluding the 5′UTR sequence of *rpoS* resulted in a non-degradable, BosR-independent *rpoS* mRNA (Figure 9), which could lead to misinterpretations that artificial induction of *rpoS* mRNA bypasses the requirement for BosR, and BosR controls *rpoS* expression at the transcriptional level, not at the post-transcriptional level.

Fur/PerR family proteins are known as DNA-binding proteins that function either as repressors or activators for gene transcription. In this study, we provide several lines of evidence that BosR, a member of the Fur/PerR family, functions as an RNA-binding protein that directly binds to the 5′UTR of *rpoS* mRNA to control RNA stability. Firstly, RNA turnover assays demonstrated that abrogation of BosR significantly impaired *rpoS* mRNA stability *in cellulo*, regardless of whether it was transcribed from the native *rpoS* promoter or from an artificial *lac* promoter (Figures 3C and 4). Secondly, the *in vitro* EMSA demonstrated that BosR binds specifically to the 5′UTR region of *rpoS* mRNA (Figures 6 and 10). Thirdly, RNA immunoprecipitation showed that BosR specifically interacts with *rpoS* mRNA *in cellulo*. It is noteworthy that the *rpoS* mRNA decay in *B. burgdorferi* is multi-phased (41). Our data showed that the primary effect of the *bosR* deletion on *rpoS* mRNA decay occurs after the first phase. Therefore, the half-life of *rpoS* mRNA decay could not be calculated using the models that describe a single exponential decay. Achieving a quantitative fit to the *rpoS* mRNA decay data requires a deeper understanding of its decay mechanism. Nonetheless, a comparison of the decay curves between the wild-type and the *bosR* mutant unequivocally illustrates the substantial impact of the *bosR* deletion on the decay of *rpoS* mRNA. More importantly, both *in vitro* and *in vivo* evidence supports the conclusion that BosR serves as an RNA-binding protein, playing a regulatory role in the modulation of *rpoS* mRNA.

It has been reported that Fur family proteins can modulate RNA decay of other genes. For example, Fur in *E. coli* represses the expression of a small RNA RyhB which in turn, facilitates decay of mRNAs of superoxide dismutase (*sodB*), succinate dehydrogenase (*sdh*), Fe–S cluster biosynthesis (*iscRSUA*), and many other genes crucial for a so-called iron-sparing response (57). However, to the best of our knowledge, BosR is the first Fur/PerR family regulator that directly binds to RNA and controls the turnover rate of *rpoS* mRNA. Interestingly, there is an emerging trend that many DNA-binding proteins can function as RNA-binding proteins (58,59). In *B. burgdorferi*, several regulators including Bpur, SpoVG and most recently, PlzA, have also been reported to be both DNA and RNA-binding regulators (60–62). Regarding BosR binding to RNA, it will be interesting to see whether the predicted N-terminal DNA-binding domain of BosR, or its C-terminal dimerization domain containing CXXC which is important for Zn binding and BosR function, is involved in RNA binding (16,27,33,63). Since the recombinant BosR used in this study was purified using a method similar to that previously reported, which has been reported containing Zn (16), it remains to be determined whether Zn is also required for BosR binding to RNA.

The *in vitro* biochemical and genetic data from this study revealed that the BosR binding site on *rpoS* mRNA overlaps with the sequence required for *rpoS* mRNA degradation (Figures 6 and 10). The genetic data show that deleting or mutating the BosR binding site resulted in a more stable *rpoS* mRNA, regardless of the presence or absence of BosR (Figures 8 and 9). This observation is quite remarkable, given that BosR-independent *rpoS* expression has not been achieved in any mutant of *B. burgdorferi* heretofore. One caveat in this study is that direct evidence showing BosR binding to the 5′UTR region and the GG sequence remains lacking. Attempts were made to perform RNA IP of BosR and mutated *rpoS* mRNA (replacement of 10nt or GG to AU mutation in 5′UTR region) by generating *Borrelia* strains carrying both BosR-HA fusion and mutated *rpoS* mRNA species. However, generating such strains was unsuccessful. Nonetheless, replacing 10nt or introducing GG to AU mutation will likely induce alterations in the folding of rpoS mRNA. Consequently, even if RNA IP experiments were successfully conducted to show that mutated *rpoS* mRNA is no longer co-immunoprecipitated with BosR, such data would not conclusively establish that BosR directly binds to these specific regions. Given that the *in cellulo* data demonstrated that replacing 10nt or introducing GG to AU mutation in the 5′UTR region led to BosR-independent *rpoS* expression, which is consistent with the *in vitro* BosR binding result (Figures 6I and 10E), we postulate that BosR binds this region *in cellulo*. Alternatively, BosR may bind a different region in *rpoS* mRNA *in cellulo*.

Overlap between the BosR binding and the *rpoS* mRNA degradation suggests that BosR stabilizes *rpoS* mRNA, possibly by occluding the ribonuclease cleavage site. Previously, the RNase III homologue in *B. burgdorferi*, *rnc*, was characterized as a potent ribonuclease that controls the turnover rate of *rpoS* mRNA (64,65). Similar to that of *E. coli*, *B. burgdorferi* RNase III recognizes and cleavages double-stranded stem RNAs (65). Thus, we hypothesize that BosR binding to the GG region in 5′UTR of *rpoS* mRNA may block RNase III binding and inhibit *rpoS* mRNA degradation. Alternatively, BosR may preferably bind to single-stranded RNA *in cellulo* and prevent double-stranded stem formation, subsequently protecting *rpoS* mRNA from RNase III cleavage. Preventing double-stranded stem formation by BosR would also release a portion of the Shine-Dalgarno (SD) sequence for ribosomal binding, thus facilitating *rpoS* translation. High levels of translation would also increase RNA stability (49,66). It is noteworthy that this study does not disapprove BosR as a DNA-binding protein, nor the BS1 and BS2 as DNA-binding sites for BosR at the *rpoS* promoter. Rather, this study demonstrates that BosR does not function as a transcription activator for *rpoS* and these binding sites are not required for *rpoS* transcriptional activation. One plausible scenario is that BosR could bind to DNA at BS1 and BS2 sites, enabling BosR to localize near *rpoS* transcripts and facilitate its binding to the 5′UTR of *rpoS* mRNA.

**Figure 11.**
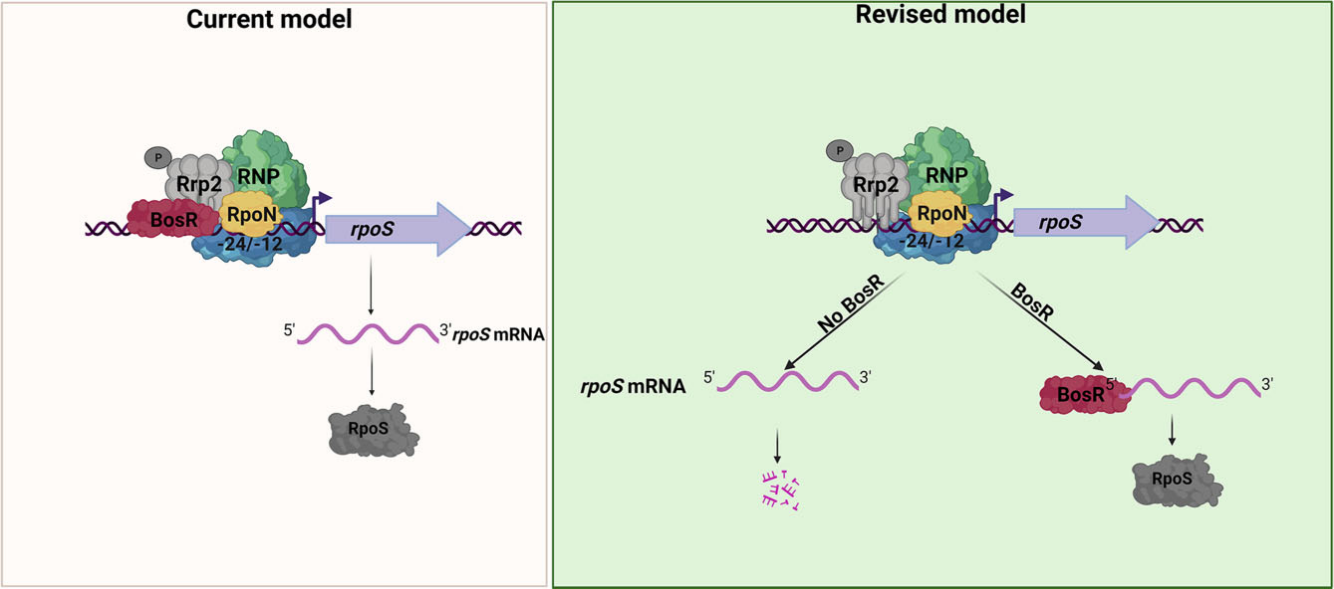
Revised model for *rpoS* expression by Rrp2, RpoN and BosR in *B. burgdorferi* In the current model, BosR functions as a transcription activator governing *rpoS* transcription at the σ^54^-type promoter. In the revised model, BosR functions as an RNA-binding protein that binds to the GG region within the 5′UTR region of *rpoS* mRNA and prevents *rpoS* mRNA degradation. The GG region is also the nucleotides required for *rpoS* mRNA degradation.

In summary, this study has identified BosR as a previously unrecognized RNA-binding protein, reshaping the established paradigm of the σ^54^–σ^S^ sigma factor cascade in *B. burgdorferi*. This finding supports a dual-layer model for RpoS regulation (Figure 11). The first layer of regulation involves the transcriptional activation of *rpoS* by Rrp2 at the σ^54^-type promoter. The second layer operates at the post-transcriptional level, where BosR binds the transcribed *rpoS* mRNA and prevents its rapid rate of degradation. Although this dual-layer regulation of *rpoS* expression has not been validated in spirochetes replicating in ticks and mammals, it is conceivable to expect that this mode of *rpoS* regulation also takes place in spirochetes replicating *in vivo*.

What is the advantage of having two layers of regulation for *rpoS* expression? Given that RpoS serves as the gatekeeper controlling the reciprocal expression of numerous *Borrelia* genes during the enzootic cycle between ticks and mammals, tight and rapid regulation of *rpoS* expression is essential (67). In the transmission phase when spirochetes encounter blood meals in nymphal ticks, it requires a quick production of RpoS to turn on *B. burgdorferi* genes needed for transmission and mammalian infection. To achieve this, the phosphorylation-dependent Rrp2 activation of RpoN allows precise and rapid activation of transcription initiation of *rpoS* at its σ^54^-type promoter. However, *rpoS* mRNA of *B. burgdorferi* processes a notably short half-life, a distinct feature different from the *rpoS* gene in other model organisms such as *E. coli* and *Salmonella*. This characteristic ensures that when RpoS needs to be OFF during the enzootic cycle, the *rpoS* mRNAs in the cell can be quickly degraded in addition to turning off *rpoS* transcription. Thus, when RpoS is needed to be ON, in addition to Rrp2-RpoN-dependent activation of *rpoS* transcription, the rapid turnover rate of *rpoS* mRNAs must be prevented, which is achieved by the presence of BosR. One interesting question raised by this study is whether, in addition to *rpoS* mRNA, BosR binds and stabilizes other RNA species in *B. burgdorferi*. Previous transcriptomic analysis revealed that while BosR- and RpoS-regulated genes largely overlap, BosR controls expressions of several genes whose expressions are independent of RpoS (31).This suggests that BosR may regulate other RNAs in addition to *rpoS* mRNA. Furthermore, this study also raises another intriguing question: do other Fur/PerR family proteins function as RNA-binding proteins?

## Supplementary Material

gkae114_Supplemental_File

## Data Availability

All data in this study has been included in the main text and the Supplementary materials.
