# Supplementary material for: A Fur family protein BosR is a novel RNA-binding protein that controls rpoS RNA stability in the Lyme disease pathogen
Source: Nucleic Acids Res. 2024 Feb 15;52(9):5320–35. doi: 10.1093/nar/gkae114 (PMC11109971; doi:10.1093/nar/gkae114)
Supplement: gkae114_Supplemental_File [file gkae114_supplemental_file.pdf]

Supplementary Table 1: List of *B. burgdorferi* strains used in the study

strain	Description	Source
**B31M**	Wild-type *B. burgdorferi*	(1)
**5A14**	Wild-type *B. burgdorferi*	(2)
**OY10H**	*bosR* mutant in B31M	(3)
**BbSR023**	*bosR* mutant in 5A14	This study
**BbSR031**	*rpoS mutant* in 5A14	This study
**BbYY028**	B31M expressing *lacp-bosR-HA*	This study
**BbJK001**	B31M expressing (BS1$^-$/BS2$^-$-*luc*) shuttle plasmid	This study
**BbJK002**	OY10H expressing (BS1$^-$/BS2$^-$-*luc*) shuttle plasmid	This study
**BbJK003**	B31M expressing (BS1$^-$/BS2$^+$-*luc*) shuttle plasmid	This study
**BbJK004**	OY10H expressing (BS1$^-$/BS2$^+$-*luc*) shuttle plasmid	This study
**BbSR123**	*rrp2* ^{G239C} mutan*t* expressing (BS1$^-$/BS2$^-$-*luc*) shuttle plasmid	This study
**BbSR124**	*rpoN* mutan*t* expressing (BS1$^-$/BS2$^-$-*luc*) shuttle plasmid	This study
**BbSR144**	B31M expressing (BS1$^+$/BS2$^+$-*luc*) shuttle plasmid	This study
**BbSR145**	OY10H expressing (BS1$^+$/BS2$^+$-*luc*) shuttle plasmid	This study
**BbSR146**	*rrp2* ^{G239C} mutant expressing (BS1$^+$/BS2$^+$-*luc*) shuttle plasmid	This study
**BbSR147**	*rpoN* mutant expressing (BS1$^+$/BS2$^+$-*luc*) shuttle plasmid	This study
**BbSR151**	*rpoS* mutant in 5A14 expressing *plac* -5'UTR *rpoS* plasmid	This study
**BbSR152**	*bosR* mutant in 5A14 expressing *plac* -5'UTR-*rpoS* plasmid	This study
**BbSR157**	*bosR* mutant expressing chromosomal *rpoSp-gfp*	This study
**BbSR158**	*rpoN* mutant expressing chromosomal *rpoSp-gfp*	This study
**BbSR161**	5A14 Wild-type expressing *rpoSp-gfp* in genome	This study
**BbSR174**	*rpoN* mutan*t* expressing chromosomal *rpoSp-gfp* was complemented with native promoter-*rpoN-HA* shuttle plasmid	This study
**BbSR177**	*rpoS* mutant in 5A14 expressing pSR079	This study

**BbSR178**	*bosR* mutant in 5A14 expressing pSR079	This study
**BbSR180**	*rpoS* mutant in 5A14 expressing pSR080	This study
**BbSR181**	*bosR* mutant in 5A14 expressing pSR080	This study
**BbSR196**	*bosR* mutant in 5A14 expressing pSR094	This study
**BbSR197**	*bosR* mutant in 5A14 expressing pSR091	This study
**BbSR198**	*bosR* mutant in 5A14 expressing pSR095	This study
**BbSR200**	*bosR* mutant in 5A14 expressing pSR092	This study
**BbSR202**	*bosR* mutant in 5A14 expressing pSR093	This study
**BbSR208**	*bosR* mutant in 5A14 expressing pSR103	This study
**BbSR222**	*rpoS* mutant in 5A14 expressing BS1^{+}/BS2^{+}-*rpoS*	This study
**BbSR223**	*rpoS* mutant in 5A14 expressing BS1^{+}/BS2^{-}-*rpoS*	This study
**BbSR224**	*rpoS* mutant in 5A14 expressing BS1^{-}/BS2^{-}-*rpoS*	This study
**BbSR225**	*bosR* mutant in AH130 expressing *plac--rpoS* ORF shuttle plasmid	This study
**BbSR227**	*bosR* mutant in AH130 expressing *plac-5'UTR*$_{flaB}$-*rpoS* ORF shuttle plasmid	This study

1. Barthold, S.W., Beck, D.S., Hansen, G.M., Terwilliger, G.A. and Moody, K.D. (1990) Lyme borreliosis in selected strains and ages of laboratory mice. *J Infect Dis*, **162**, 133-138.
2. Purser, J.E. and Norris, S.J. (2000) Correlation between plasmid content and infectivity in *Borrelia burgdorferi Proc Natl Acad Sci U S A*, **97**, 13865-13870.
3. Ouyang, Z., Kumar, M., Kariu, T., Haq, S., Goldberg, M., Pal, U. and Norgard, M.V. (2009) BosR (BB0647) governs virulence expression in Borrelia burgdorferi. *Molecular microbiology*, **74**, 1331-1343.

Supplementary Table 2: List of plasmids used in the study

plasmid	Description	Antibiotic marker
**BS1^{+}/BS2^{+}-*luc***	pJD48 containing 250 bp upstream region of *rpoS*	*Gent*^{R}
**BS1^{+}/BS2^{-}-*luc***	pJD48 containing 95 bp upstream region of *rpoS*	*Gent*^{R}
**BS1^{-}/BS2^{-}-*luc***	pJD48 containing 78 bp upstream region of *rpoS*	*Gent*^{R}
**pYY012**	shuttle plasmid expressing *plac-bosR-3'HA* fusion	*strep*^{R}
**pSR069**	shuttle plasmid expressing *plac-5'*UTR$_{rpoS}$-*rpoS*	*strep*^{R}
**pSR074**	Suicidal plasmid used for replacing *rpoS* with codon optimized *B. burgdorferi gfp*	*strep*^{R}
**pSR079**	shuttle plasmid carrying minimal promoter- UTR$_{flaB}$-*rpoS*	*Rif*^{R} for *E. coli* and *Blast*^{R} for *B. burgdorferi*
**pSR080**	shuttle plasmid carrying minimal promoter- UTR$_{rpoS}$-*rpoS*	*Rif*^{R} for *E. coli* and *Blast*^{R} for *B. burgdorferi*
**pSR094**	shuttle plasmid carrying minimal promoter-30 bp Wild type-20 bp UTR$_{flaB}$-*rpoS*	*Rif*^{R} for *E. coli* and *Blast*^{R} for *B. burgdorferi*

**pSR091**	shuttle plasmid carrying minimal promoter-30 bp UTR$_{flaB}$-20 bp UTR$_{rpoS}$-*rpoS*	*Rif*^{R} for *E. coli* and *Blast*^{R} for *B. burgdorferi*
**pSR093**	shuttle plasmid carrying minimal promoter - 40 bp UTR$_{rpoS}$ -10 bp UTR$_{flaB}$ -*rpoS*	*Rif*^{R} for *E. coli* and *Blast*^{R} for *B. burgdorferi*
**pSR092**	shuttle plasmid carrying minimal promoter-30 bp UTR$_{rpoS}$ -10 bp UTR$_{flaB}$ -10 bp UTR$_{rpoS}$ -*rpoS*	*Rif*^{R} for *E. coli* and *Blast*^{R} for *B. burgdorferi*
**pSR095**	shuttle plasmid carrying minimal promoter-UTR$_{rpoS}$ containing a point mutation in SD-*rpoS*	*Rif*^{R} for *E. coli* and *Blast*^{R} for *Bb*
**pSR103**	shuttle plasmid carrying minimal promoter-UTR$_{rpoS}$ containing GG-AT mutation in SD-*rpoS* ORF	*Rif*^{R} for *E. coli* and *Blast*^{R} for *B. burgdorferi*
**pSR104**	shuttle plasmid carrying minimal promoter-UTR$_{rpoS}$ containing CC-AT mutation-*rpoS* ORF	*Rif*^{R} for *E. coli* and *Blast*^{R} for *B. burgdorferi*
**BS1^{+}/BS2^{+}-*rpoS***	pBSV2B containing *rpoS* 250 bp upstream region -*rpoS ORF*	*Rif*^{R} for *E. coli* and *Blast*^{R} for *B. burgdorferi*
**BS1^{+}/BS2^{-}-*rpoS***	pBSV2B containing *rpoS* 95 bp upstream region -*rpoS ORF*	*Rif*^{R} for *E. coli* and *Blast*^{R} for *B. burgdorferi*
**BS1^{-}/BS2^{-}-*rpoS***	pBSV2B containing *rpoS* 78 bp upstream region - *rpoS ORF*	*Rif*^{R} for *E. coli* and *Blast*^{R} for *B. burgdorferi*
**pSR115**	shuttle plasmid expressing *plac-5'UTR*$_{flaB}$-*rpoS* ORF	*strep*^{R}
**pOY110**	shuttle plasmid expressing *plac-rpoS* ORF	*strep*^{R}

Supplementary Table 3: List of cloning primers used in the study

Primer	Sequence (5'-3')*
**BS1^{+}/BS2^{+}-*luc* F**	GATACCATGGTTTTTGAAGATATGCTTTGCGAAC
**BS1^{+}/BS2^{+}-*luc* R**	GATACATATGAGTTATTATATTTTCTCCCCTTTC
**BS1^{+}/BS2^{-}-*luc* F**	GATACCATGGAAATTAAATTGGCACAGTTTTTGCA
**BS1^{-}/BS2^{-}-*luc* F**	GATACCATGGTGGCACAGTTTTTGCATGGAAA
**pYY012F**	GATACATATGAACGACAACATAATAGACGTAC
**pYY012R**	GATAAGATCTATCAAGCGTAATCTGGAACATCGTATGGGTATAAAGTGATTTCCTTGTTCTCATC
**pSR069F**	GATACATATGAGTAAAAACTTAATCACAATATTC
**pSR069R**	GATAAGATCTTTAATTTATTTCTTCTTTTAATTTT
**pSR074Oligo-1**	GGGCTGCAGCCCGGGGGATCCCTATTTGTATAGTTCATCCATGCCA
**pSR074Oligo-2**	TATAATAACTATGAGTAAAGGAGAAGAACTTTTCA
**pSR074Oligo-3**	CTTTACTCATAGTTATTATATTTTCTACCC
**pSR074oligo-4**	TCTAGAACTAGTGGATCCTAAAATAATAGATATTAAAGAAAT

**pSR079F**	GATAGAGCTCTTTTAAATTAAATTGGCACAGTTTTTGCATGGAAATTAAGTAAGGATTTGCCAAAGTCAGAAATTTAAATTTTATCATGGAGGAATGATATATGAACATATTTAGTAATGAGGATTT
**pSR07R**	GATAGCATGCTTAATTTATTTCTTCTTTTAATTT
**pSR080F**	GATAGAGCTCTTTTAAATTAAATTGGCACAGTTTTTGC
**pSR094F**	GATAGAGCTCTTTTAAATTAAATTGGCACAGTTTTTGCATGGAAATTAAGTAAGGATTTGCCAAAGTCAGAAATTTAAATTGGGGAGAAAATATAATAACT
**pSR091F**	GATAGAGCTCTTTTAAATTAAATTGGCACAGTTTTTGCATGGAAATTAAGTAGTAAAAACTTAATCACAATATTCAAGAAATTATCATGGAGGAATGATAT
**pSR093F**	GATAGAGCTCTTTTAAATTAAATTGGCACAGTTTTTGCATGGAAATTAAGAGTAAAAACTTAATCACAATATTCAAGAAAGGGGAGAAAAGCCATGATAT
**pSR092F**	GATAGAGCTCTTTTAAATTAAATTGGCACAGTTTTTGCATGGAAATTAAGTAGTAAAAACTTAATCACAATATTCAAGAAAATGGAGGAATTATAATAACT
**pSR095F**	GATAGAGCTCTTTTAAATTAAATTGGCACAGTTTTTGCATGGAAATTAAGTAGTAAAAACTTAATCACAATATTCAAGAAAGGGGAGGAAATATAATAACT
**pSR103F**	GATAGAGCTCTTTTAAATTAAATTGGCACAGTTTTTGCATGGAAATTAAGTAGTAAAAACTTAATCACAATATTCAAGAAAATGGAGAAAATATAATAACT
**pSR104F**	TATTCATTATCTTGAAAAAGAATATTCTCTG
**pSR104R**	ATTTTTATATACTGAGCAGGAGATAG

**BS1^{+}/BS2^{+}-** ***rpoS*** **F**	GATAGAGCTCTTTTTGAAGATATGCTTTGCGAAC
**BS1^{+}/BS2^{-}-** ***rpoS*** **F**	GATAGCATGCTTAATTTATTTCTTCTTTTAATTT
**BS1^{-}/BS2^{-}-** ***rpoS*** **F**	GATAGAGCTCTGGCACAGTTTTTGCATGGAAATTA

* Restriction sites are indicated by underlining

Supplementary Table 4: List of Primers/Oligo nucleotides used for RNA EMSA

**Oligo**	**Sequence (5’-3’)***
**RNA-1F**	TAATACGACTCACTATAGGGAAGTAGTAAAAACTTAATCACAATATTC
**RNA-1R**	TTCTGTTAAATATTTATTTATT
**RNA-2F**	TAATACGACTCACTATAGGGGAAGAAAAATCGCCCAAAAAAGAAG
**RNA-2R**	TTAATTTATTTCTTCTTTTAATTTT
**RNA-3R**	CTTACTGATTTTAAATATATG
**RNA-4F**	TAATACGACTCACTATAGGGAGAACACAAGCTAATTACTCACG
**RNA-5R**	TTCATAGTTATTATATTTTCTC
**RNA-UTR*flaB***	TAATACGACTCACTATAGGGAAGGATTTGCCAAAGTCAGAAATTTAA
**Wild-type StemRNA (S)**	TAATACGACTCACTATAGGGCAATATTCAAGAAAGGGGAGAAAAAATTCCCTATCTTGAAAAA
**Wild-type StemRNA (A)**	ATTATGCTGAGTGATATCCCGTTATAAGTTCTTTCCCCTCTTTTTTAAGGGATAGAACTTTTT
**GG-AT Mut StemRNA (S)**	TAATACGACTCACTATAGGGCAATATTCAAGAAAATGGAGAAAAAATTCCCTATCTTGAAAAA
**GG-AT Mut StemRNA (A)**	ATTATGCTGAGTGATATCCCGTTATAAGTTCTTTTACCTCTTTTTTAAGGGATAGAACTTTTT
**CC-AT Mut StemRNA (S)**	TAATACGACTCACTATAGGGCAATATTCAAGAAAGGGGAGAAAAAATTCATTATCTTGAAAAA
**CC-AT Mut StemRNA (A)**	ATTATGCTGAGTGATATCCCGTTATAAGTTCTTTCCCCTCTTTTTTAAGTAATAGAACTTTTT

**54 nt 23S rRNA stem (S)**	TAATACGACTCACTATAGGGGATGGAAGATAAAAATATGGTCAAAGGCTTTGGCCATATTTTTGTCTTCCT
**54 nt 23S rRNA stem (A)**	ATTATGCTGAGTGATATCCCCTACCTTCTATTTTTATACCAGTTTCCGAAACCGGTATAAAAACAGAAGGA

*The underline shows the minimum T7 promoter sequence needed for transcription

Supplementary figures

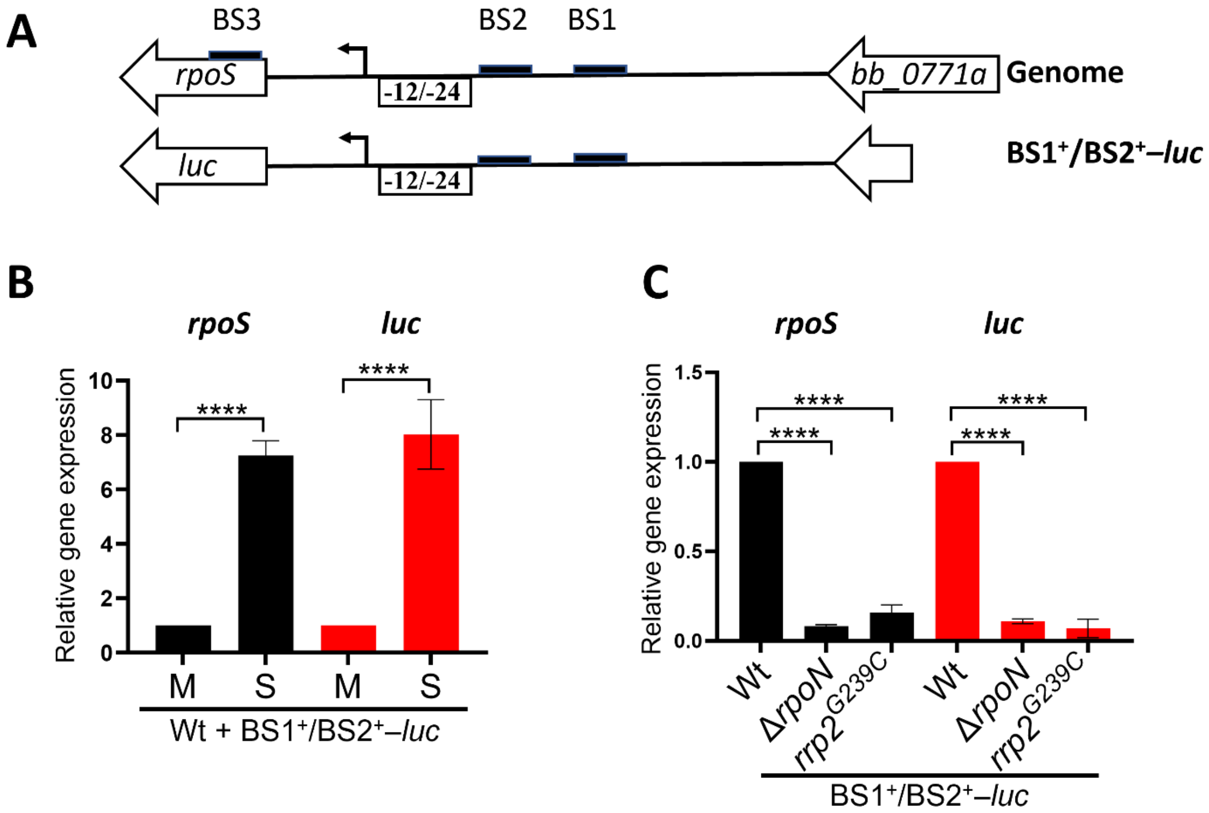

Supplementary Figure 1. **Establishing a luciferase (*luc*) reporter system for studying *rpoS* promoter activity.** (**A**) **Schematic representation of the BS1$^+$/BS2$^+$-*luc* reporter construct**. The top diagram represents the organization of *rpoS* gene in the chromosome in *B. burgdorferi* genome. The lower diagram depicts the DNA fragment containing a *luc* gene driven by the *rpoS* promoter (BS1$^+$/BS2$^+$-*luc*). The black boxes indicate the location of three putative BosR binding sites (BS1, BS2 and BS3) identified previously. The open box labeled with -12/-24 represents the major *rpoS* promoter (a σ^{54}-type promoter). The arrow indicates the transcription start site (TSS) of *rpoS*. (**B**) and (**C**), **Quantitation of native *rpoS* and *luc* reporter gene expression by qRT-PCR.** Wild-type *B. burgdorferi* strain B31 (Wt), the *rpoS* mutant, or *rrp2*^{G}*239*^{C} mutant carrying BS1$^+$/BS2$^+$-*luc* reporter plasmid, were cultured in BSK-II medium at 37° and harvested at either mid-log (**M**) or stationary (**S**) phases. RNAs were extracted and subjected to qRT-PCR analyses for expressions of both native *rpoS* and *luc* and are plotted as their respective relative gene expression. Black bars represent chromosomal *rpoS* expression and red bars represents *luc* expression, respectively. For **Figure 1B**, the expression levels of both *rpoS* and *luc* isolated from a mid-log culture was taken as 1.0. For **Figure 1C**, spirochetes were harvested at stationary phases, and the levels of *rpoS* and *luc* transcripts in wild-type *B. burgdorferi* were taken as 1. The bars represent the mean values of three independent experiments, and the error bars represent the standard deviation. ****, $p < 0.00001$ respectively using one-way Anova.

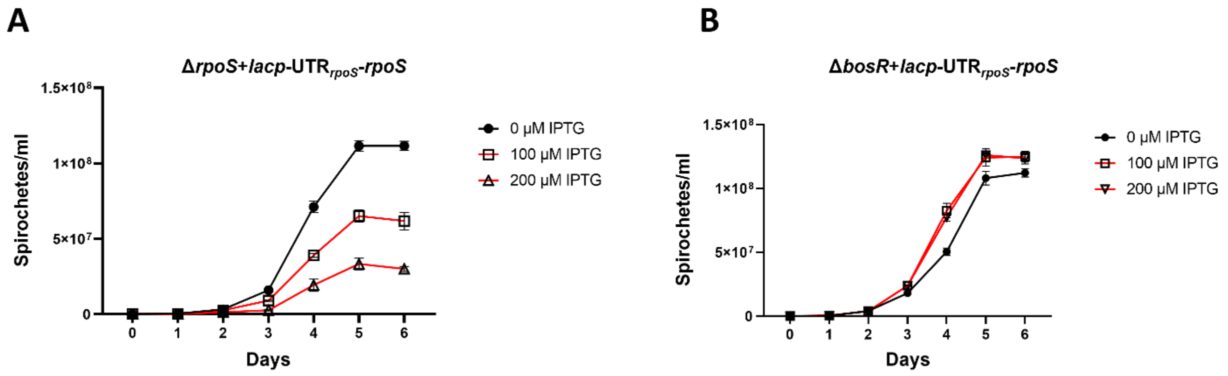

Supplementary figure S2. Growth curve studies. Growth curves of strains Δ*rpoS*+*lacp*-UTR$_{rpoS}$-*rpoS* (**A**) and Δ*bosR*+*lacp*-UTR$_{rpoS}$-*rpoS* (**B**) grown for 6 days in the presence of (100 µM and 200 µM) or absence (0 µM) of IPTG. Values in the growth curves represent the mean cell counts ± standard deviations (SD) from three independent cultures.

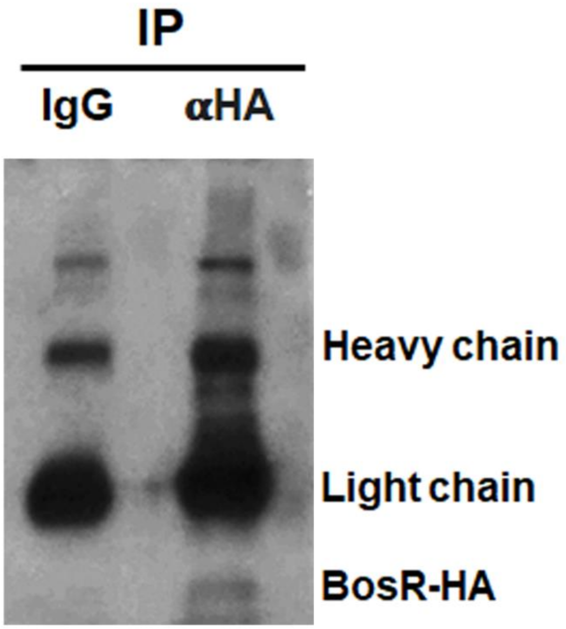

Supplementary Figure S3. **Immunoprecipitation of BosR-HA**. Spirochetes grown in the presence of 100 μg/ml of IPTG were subjected to immunoprecipitation using anti-HA or normal IgG. Precipitated samples were then subjected to immunoblotting using anti-BosR monoclonal antibody.

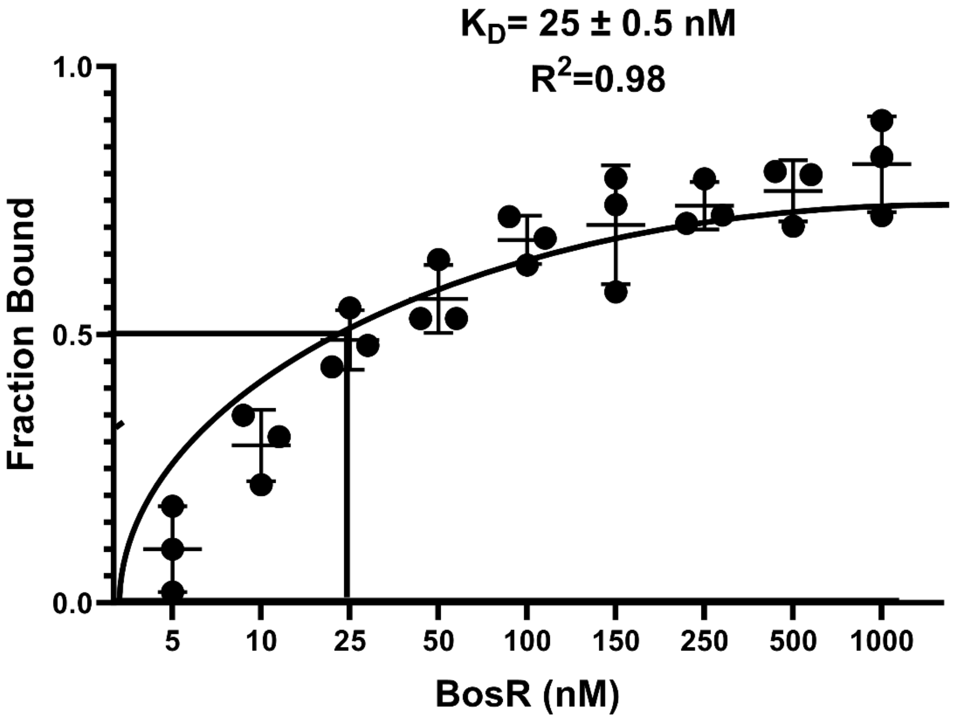

Supplementary Figure S4: **Calculation of Dissociation Constant of BosR binding to *rpoS* mRNA**. The Dissociation Constant (K_D) of BosR binding to *rpoS* mRNA was determined from its binding isotherm by curve-fitting using nonlinear regression. The mean K_D value from three repeat experiments was calculated as 25 ± 0.5 nM. Each dot represents individual values from three independent experiments and error bar represents their respective standard deviation.

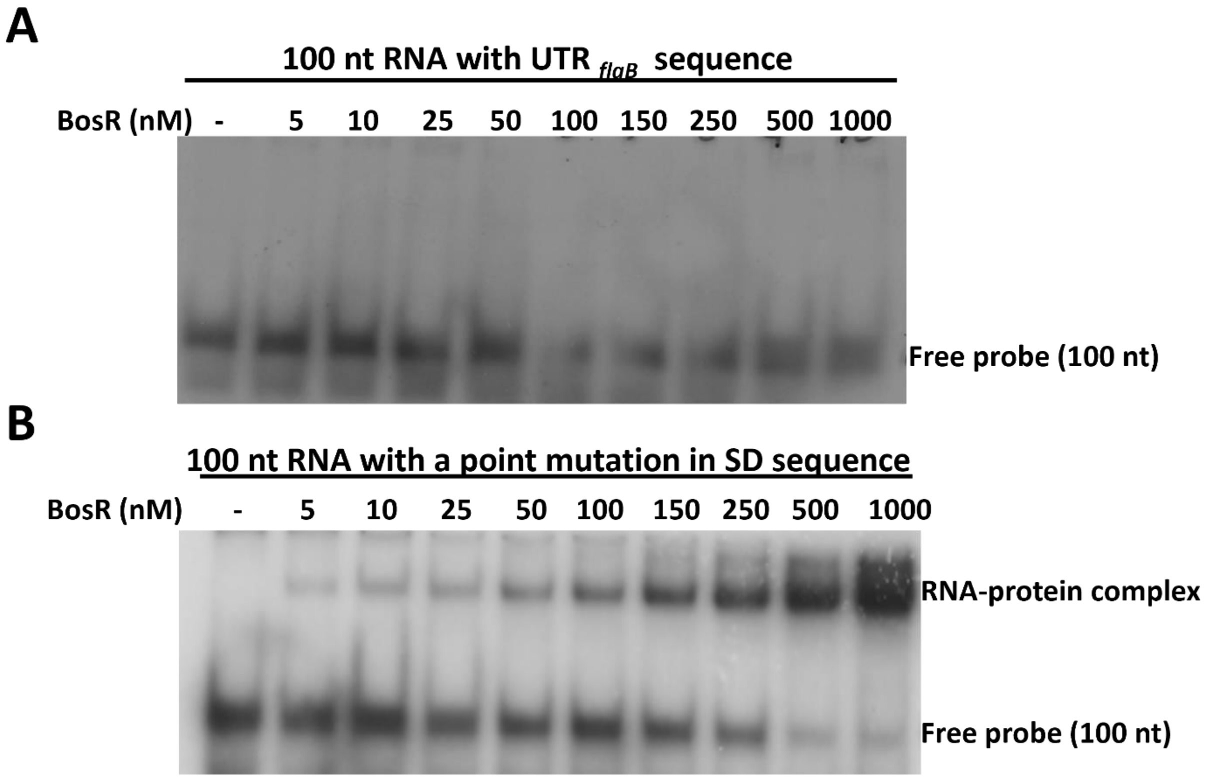

Supplementary figure S5. RNA EMSA. RNA EMSA using BosR protein and various *rpoS* RNA species. For all the EMSA 50 nM concentration of respective RNA were incubated with varying concentration of BosR as mentioned on the top each figure and separated on a native TBE gel. RNA protein complex and free probes with respective sizes are mentioned in the figure. (**A**) RNA EMSA of BosR protein and *flaB* UTR RNA species. (**B**) RNA EMSA of BosR protein and *rpoS* UTR RNA species containing single point mutation in shine-Dalgarno sequence.
